# LMP1 and EBNA2 constitute a minimal set of EBV genes for transformation of human B cells

**DOI:** 10.3389/fimmu.2023.1331730

**Published:** 2023-12-19

**Authors:** Jingwei Zhang, Thomas Sommermann, Xun Li, Lutz Gieselmann, Kathrin de la Rosa, Maria Stecklum, Florian Klein, Christine Kocks, Klaus Rajewsky

**Affiliations:** ^1^ Max-Delbrück-Center for Molecular Medicine in the Helmholtz Association (MDC), Immune Regulation and Cancer, Berlin, Germany; ^2^ Laboratory of Experimental Immunology, Institute of Virology, Faculty of Medicine and University Hospital Cologne, University of Cologne, Cologne, Germany; ^3^ German Center for Infection Research (DZIF), Partner Site Bonn-Cologne, Cologne, Germany; ^4^ Max-Delbrück-Center for Molecular Medicine in the Helmholtz Association (MDC), Immune Mechanisms and Human Antibodies, Berlin, Germany; ^5^ Berlin Institute of Health (BIH) at Charité, Center of Biological Design, Berlin, Germany; ^6^ Experimental Pharmacology and Oncology (EPO) Berlin-Buch GmbH, Berlin, Germany; ^7^ Center for Molecular Medicine Cologne (CMMC), University of Cologne, Cologne, Germany

**Keywords:** B cell lymphoma, lymphomagenesis, Epstein-Barr virus (EBV), EBV latent genes, Epstein-Barr nuclear antigen 2 (EBNA2), latent membrane protein 1 (LMP1), lymphoblastoid cell line (LCL), transduction of human primary B cells

## Abstract

**Introduction:**

Epstein-Barr virus (EBV) infection in humans is associated with a wide range of diseases including malignancies of different origins, most prominently B cells. Several EBV latent genes are thought to act together in B cell immortalization, but a minimal set of EBV genes sufficient for transformation remains to be identified.

**Methods:**

Here, we addressed this question by transducing human peripheral B cells from EBV-negative donors with retrovirus expressing the latent EBV genes encoding Latent Membrane Protein (LMP) 1 and 2A and Epstein-Barr Nuclear Antigen (EBNA) 2.

**Results:**

LMP1 together with EBNA2, but not LMP1 alone or in combination with LMP2A was able to transform human primary B cells. LMP1/EBNA2-immortalized cell lines shared surface markers with EBV-transformed lymphoblastoid cell lines (LCLs). They showed sustained growth for more than 60 days, albeit at a lower growth rate than EBV-transformed LCLs. LMP1/EBNA2-immortalized cell lines generated tumors when transplanted subcutaneously into severely immunodeficient NOG mice.

**Conclusion:**

Our results identify a minimal set of EBV proteins sufficient for B cell transformation.

## Introduction

EBV or Human Herpesvirus 4 is a gamma herpesvirus, closely related to non-human, primate lymphocryptoviruses that infect chimpanzees and rhesus monkeys (1). EBV is an ancient virus that has co-evolved with humans (2), and has become one of the most widespread human pathogens, present in close to 95% of the adult human population today (3). It infects epithelial cells and B cells, and establishes lifelong, latent infections that are associated with a wide variety of diseases and cancers (2, 4). In healthy EBV-infected individuals, resurgence of the virus is actively suppressed by T cells and innate lymphocytes (4–6). However, under conditions of immune suppression pathologies can arise such as post-transplant lymphoproliferative diseases (PLDs) and EBV-associated B lymphomas (4, 7). Other pathologies, such as symptomatic primary infection (infectious mononucleosis), haemophagocytic lymphohistiocytosis, and possibly, multiple sclerosis (8) may be caused by an overactivated immune response that is unable to clear the virus (4, 7, 9).

EBV is estimated to contribute to 1-2% of the global tumor burden, with about 200,000 cases per year (10–12). EBV’s oncogenicity is related to its ability to persist in the host by establishing a cellular reservoir in long-lived memory B cells (1, 4, 5, 7, 13). After entering the body through saliva and oropharyngeal epithelium, EBV infects naïve tonsillar B cells and causes their activation, proliferation, and protection from cell death via a transcriptional gene expression program referred to as the ‘latency III’ or ‘growth latency’ transcription program. This comprises two non-coding EBV-encoded small RNAs (EBERs), at least 44 miRNAs, and eight EBV latent genes (coding for six EBV nuclear antigens (EBNAs) and two latent membrane proteins (LMP1 and LMP2) (1, 7, 13, 14).

According to the Germinal Center Model of EBV persistence (14, 15), following activation by ‘latency III’, infected B cell blasts enter the germinal center. Here, expression of the latent EBV proteins EBNA1, LMP1 and LMP2 (latency II) ensures their survival and viral access to the B memory cell pool. In long-lived memory B cells, EBV can persist without viral protein expression in resting B cells (latency 0) or with expression of only EBNA1, which is required for replication and maintenance of episomal viral genomes in homeostatically dividing memory B cells (latency I). The latent EBV gene expression programs are tailored to normal B cell developmental differentiation stages from immunoblastic to memory B cells, and are also preserved in EBV-associated B lymphomas, with some diffuse large B cell lymphomas expressing latency III, Hodgkin’s lymphoma expressing latency II, and Burkitt lymphoma expressing latency I genes (1, 7, 14).

In cell culture, EBV can readily transform resting human B cells into indefinitely growing lymphoblastoid cell lines (LCLs) (1, 7, 13, 14). Established LCLs express the latency III program and resemble infected B cells from infectious mononucleosis or PLD patients or EBV-associated B cell lymphomas that arose under immune suppression. The importance of individual EBV latent genes for B cell immortalization has been extensively studied, most recently genetically, using recombinant viruses (16). From these studies, as well as numerous earlier works, the transcriptional transactivator EBNA2 emerged as a key factor that is essential for all phases of the immortalization process (17–19). EBNA2 is sufficient to reprogram B cells from a resting, energetically quiescent to an activated, proliferating state, and both, the initial metabolic activation and the subsequent cell cycle progression require the presence of EBNA2 protein (16, 20). EBNA2 subverts Notch signaling (21) and transcriptionally activates a cascade of primary and secondary target genes, most prominently the cellular proto-oncogene MYC and its targets, and the viral promoters that control LMP1 and LMP2 expression (22).

Similar to Notch, EBNA2 does not bind DNA directly, but engages cellular transcription factors, and influences their function. Through a central ‘adapter’ region EBNA2 binds the Notch effector RBPJ (RBPJk/CBF1), a sequence-specific, conserved ubiquitous repressor that occupies super enhancers in B cells and represses target genes (19). EBNA2 relieves this repression, thereby activating RBPJ target genes, including MYC. In parallel, through its N-terminal domain, EBNA2 recruits Early B Cell Factor 1 (EBF1/OLF-1/COE1) (23), a pioneer transcription factor that specifies and maintains B cell lineage (24). EBF1 binding by EBNA2 is necessary for full oncogenic hyperactivation of MYC, to promote metabolic processes linked to cell cycle progression, thereby maintaining B cells in their EBV-driven, proliferating, immortalized state (20).

LMP1 is regarded as the major EBV oncogene (25). It subverts signaling by the TNF-receptor family member CD40, leading to constitutive activation of pro-proliferative and pro-survival signals, through several TRAF-dependent signaling pathways including NF-kB, PI3-kinase, the MAP kinases JNK, p38 and ERK and IRF7 (13, 25–30). We previously showed that in T cell-deficient mice, transgenic expression of LMP1 in B cells causes a lymphoproliferative disease that leads to the development of monoclonal B cell lymphomas (31). These LMP1-driven lymphomas acquire *in vivo* secondary oncogenic mutations that aberrantly activate either *Ebf1* or the NF-kB subunit *Rel* (32).

We subsequently showed that the coexpression of a single viral gene, LMP1, and a single cellular host gene, EBF1, increased survival and proliferation of human tonsillar B cells in culture, and was sufficient to recapitulate EBV-mediated transformation of B cells in mice (32). In contrast to this, MYC-driven malignant transformation of mature mouse B cells requires inhibition of both intrinsic apoptosis and p53 activity by overexpression of at least two additional factors, such as BCL6/BCL2 or BMI1/BCLXL or BMI1/MCL1 (33, 34). Based on these findings, we reasoned that combining the pro-proliferative and pro-survival activities induced by LMP1 (35) with the full induction of oncogenic MYC levels by EBNA2 (through its cellular effectors RBPJ and EBF1) (20) might be sufficient to immortalize B cells. In the present paper, we set out to test the hypothesis that EBNA2 and LMP1 represent a minimal set of EBV latent genes for the growth transformation of human primary B cells.

## Materials and methods

### Study individuals and sample collection

Samples from EBV-negative donors were obtained under a study protocol approved by the Institutional Review Board of the University of Cologne and respective local Institutional Review Boards (study protocol 16-054). All participants provided written informed consent and were recruited as outpatients. Sites of recruitment was the Institute of Virology at the University Hospital Cologne.

### Isolation and culture of B cells

CD19+ B cells were isolated from PBMCs by positive selection using CD19 Microbeads (Miltenyi, Cat. No. 130-050-301) and seeded at concentration of 0.5 million/ml. B cells were cultured either with recombinant human CD40L trimers (2 µg/ml; CD40L-Tri; purified in-house) (36) and IL-21 (100 ng/ml; Biozol, Cat. No. BLD-571208) or on a CD40L/IL21-expressing feeder cell line (33). Every 4-5 days the activation stimulus was replenished.

### Retroviral transduction and infection with EBV

The coding sequence (CDS) of EBV strain B95-8 latent proteins EBNA2, LMP1 and LMP2A was inserted into an MSCV backbone based on pMIG (MSCV IRES GFP; Addgene plasmid #9044). The EBNA2 CDS was optimized for protein expression in mammalian cells on the transcriptional, mRNA and translational level and custom synthesized (GeneOptimizer Software, GeneArt Gene Synthesis Services, ThermoFisher) (37). Transfection of 293T cells, retrovirus production and B cell transduction was performed as described (33). For infection with EBV wildtype strain B95-8 (38, 39), human peripheral B cells were infected 2 days after activation with supernatant containing virus for 3 hours and seeded at 0.5 million cells/ml.

### Flow cytometry and Western blots

Cells were stained with fluorophore-conjugated antibodies and resuspended in FACS buffer containing a Live/Dead staining-dye (DAPI, PI, or LIVE/DEAD Fixable Near-IR Dead Cell Stain Kit, ThermoFisher SCIENTIFIC, Cat. No. L34976) and counting beads (CountBright absolute counting beads, Life Technologies, Cat. No. C36950), then analyzed on a LSRFortessa (BD Biosciences) using FlowJoTM v10.6.2 Software (BD Biosciences). For DNA content analysis, cells were labeled with LIVE/DEAD Fixable Near-IR Dead Cell Stain Kit (ThermoFisher SCIENTIFIC, Cat. No. L34976), fixed with 1X Cytofix/Cytoperm buffer (BD, Cat. No. 554722) and stained with DAPI. The DAPI channel was acquired on a linear scale. Protein extraction and Western Blots were performed with standard procedures.

### Analysis of cell growth

At the indicated time points, cells were collected and resuspended in 100 µl FACS buffer and analyzed by flow cytometry in the presence of 1 µl CountBright Absolute counting beads for flow cytometry (Invitrogen, Cat. No. C36950) until 400 to 500 beads were acquired (as gated by size and fluorescence intensity). The concentration of cells was determined as described by the manufacturer. From this value, the total cell number for each culture was calculated based on the total volume of the cell culture and the recorded split ratio (since day 3). Cells were split at a ratio of up to 1:2, when necessary, as judged by cell density and medium pH, and only when they reached more than 1 million cells/ml (resulting in newly split cells at a concentration of 1-2 million/ml). Cells were pipetted up and down softly to break up larger cell clumps. Between day 25 and 37 (the time window when LMP1 single-positive cells ceased to grow), cultures were not, or only rarely, split. Cell viability was measured by trypan blue dye exclusion in an automated cell counter (TC20, BIO-RAD) with particles with a diameter below 5 μm excluded.

### Tumor growth in xenotransplanted NOG mice

Mouse experiments were approved by the Landesamt für Gesundheit und Soziales Berlin (Reg 0010/19) and complied with the German Animal Welfare Act. B cell lines frozen 60 days after transduction were thawed and cultured for 2 weeks. 100 μl containing 5 million cells in 50% MatriGel (Corning, Cat. No. 356234) were injected subcutaneously into female, 2-month-old NOG mice (NOD.Cg-*Prkdc^scid^ Il2rg^tm1Sug^*/JicTac; Taconic Biosciences). Three times per week the tumor volume was measured and calculated using the formula 0.5 a × b^2^, where a and b are the long and short diameters of the tumor, respectively. After reaching a tumor volume of ≥ 1.5 cm^3^ animals had to be sacrificed (humane endpoint).

### Statistical analyses

Means, medians and statistical significance were calculated using GraphPad Prism 8 (version 8.4.3) by the tests and *p* value corrections indicated in the figure legends. We followed Prism’s recommendations when prompted. We used the New England Journal of Medicine *p* value style, which shows three digits. A *p* > 0.05 was considered not significant (ns).

Further details and materials can be found in **Supplementary Material**.

## Results

### Expression of EBV latent genes in primary human B cells

Human primary B cells are refractory to transduction with existing amphotropic retrovirus systems or with retrovirus pseudotyped with vesicular stomatitis virus G glycoprotein (VSV-G) (33, 40, 41). Therefore, we turned to a strategy which employs the envelope protein of Gibbon ape leukemia virus (GaLVenv) (42) to achieve efficient viral transduction of non-malignant primary human germinal center B cells *ex vivo* (33). GaLVenv-pseudotyped retrovirus (GaLVenv-RV) efficiently transduced both naïve and memory B cells isolated from peripheral blood mononuclear cells (PBMCs) (**Supplementary Figures 1A–C**).

**Figure 1:**
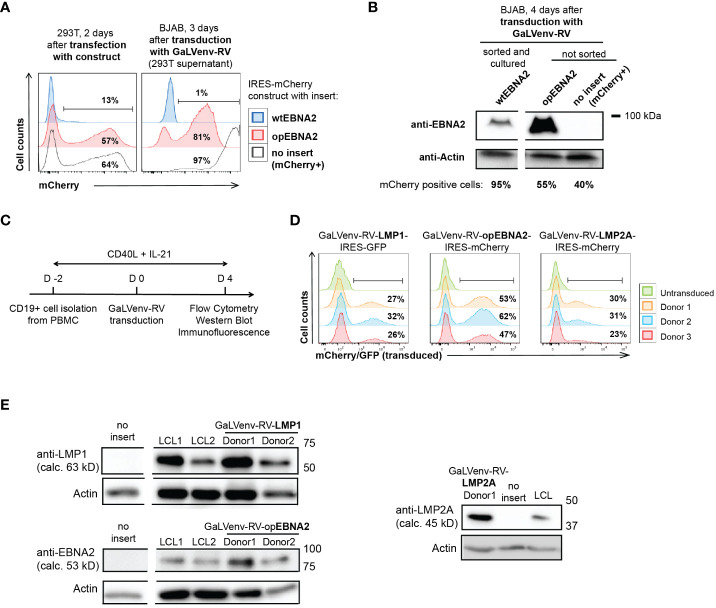
GaLVenv-Retroviral transduction enables expression of the EBV latent protein LMP1, LMP2A and EBNA2 in human primary B cells. **(A, B)** Expression of EBNA2 after RNA and codon optimization (37). **(A)** (Left) mCherry reporter levels measured by flow cytometry after transfection of 293T packaging cells with a bicistronic retroviral IRES-mCherry plasmid. mCherry expression was elevated for optimized EBNA2 (opEBNA2) compared to wildtype EBNA2 (wtEBNA2), indicating increased mRNA expression. (Right) mCherry reporter-positive BJAB cells measured 3 days after transduction with 293T cell supernatants containing GaLVenv-pseudotyped retrovirus (GaLVenv-RV). The increase in reporter-positive cells reflects elevated retroviral titers for opEBNA2 compared to wtEBNA2. **(B)** Western blot showing enhanced EBNA2 protein levels four days after transduction of BJAB cells. wtEBNA2-expressing cells were pre-enriched by sorting and expanding of mCherry-positive cells. The percentage of mCherry-positive cells is indicated. **(C)** Experimental set up for panel **(D, E)**, and **Supplementary Figures 1D, F**, CD19-positive B cells were isolated from PBMCs from three untested healthy donors and stimulated for two days with soluble CD40-ligand trimer and human IL-21, then transduced with GaLVenv-RV (day 0). Four days after transduction, cells were collected for analysis. **(D, E)** Expression of EBV latent proteins in human primary B cells. **(D)** GFP or mCherry reporter-positive cells measured by flow cytometry after transduction with GaLVenv-RV carrying open reading frames of LMP1, LMP2A, and opEBNA2 individually fused to IRES-mCherry or -GFP. Gating is indicated. **(E)** Western Blots showing expression of LMP1, LMP2A and opEBNA2 proteins four days after transduction in unsorted cell populations containing mixtures of transduced and non-transduced cells. Cells transduced with GFP or mCherry were used as negative controls (no insert); LCL cell lysate was used as positive and actin as loading control. Closest molecular weight markers flanking the detected band are indicated at the right (not drawn to scale). EBNA2 migrates at 70-90 kD in SDS-PAGE (18). calc., calculated.

Using this transduction system, we set out to investigate how expression of the EBV latent genes EBNA2, LMP1 and LMP2A contributes to the growth transformation of human primary B cells. However, EBNA2 was poorly expressed from retroviral constructs, both in mouse (32) and human B cells (**Figure 1A**). To overcome this problem, we *de novo* synthesized the coding sequence of EBNA2 after multiparameter expression optimization (37). Optimized EBNA2 (opEBNA2) showed dramatically improved mCherry reporter expression over wildtype EBNA2 in 293T retroviral packaging cells, leading to higher virus titers (as measured by transducing BJAB cells), and to higher EBNA2 protein expression levels in transduced BJAB cells (**Figures 1A, B** **;** **Supplementary Figure 1D**).

We then generated retroviruses carrying coding sequences for opEBNA2, LMP1 and LMP2A, and transduced PBMC-derived B cells in the presence of CD40 ligand (CD40L) trimers and IL-21 (**Figure 1C**). (In our hands, transduction of quiescent B cells with GaLV-pseudotyped retrovirus was ineffective (**Supplementary Figure 1E**), although it has been reported previously for GaLV-pseudotyped lentivirus (42)). Expression of the three EBV latent proteins was confirmed by monitoring mCherry reporter expression by flow cytometry, and by Western blot and immunofluorescence (**Figures 1D, E** **;** **Supplementary Figure 1F**). As expected from the literature, LMP1 and LMP2A predominantly localized to the perinuclear region in the cytoplasm (43–45), whereas EBNA2 was observed in *punctae* in the nucleus (46, 47) (**Supplementary Figure 1F**). The localization patterns and expression levels appeared comparable to those detected in LCLs that were generated in parallel by infecting pre-activated, primary B cells with EBV.

Taken together, we successfully established a transduction and expression system for primary human B cells that is suitable to study the immortalizing and growth-transforming properties of the EBV latent proteins EBNA2, LMP1 and LMP2A.

### Coexpression of LMP1 and EBNA2 confers long term growth on human primary B cells

**Figure 2:**
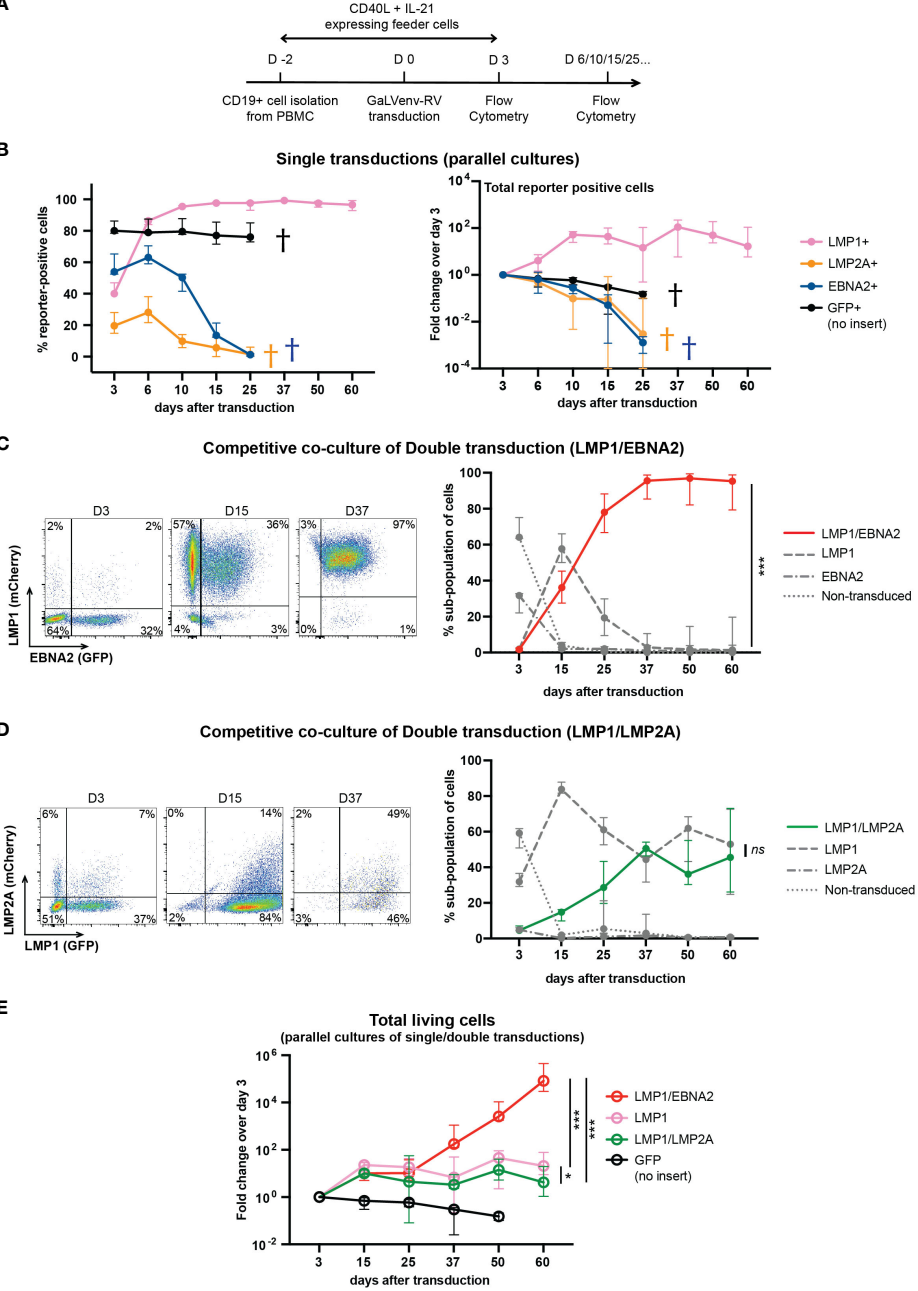
LMP1/EBNA2-coexpressing human peripheral B cells show long-term proliferative outgrowth in cell culture. **(A)** Experimental scheme. **(B)** Percentage (left panel) and total (right panel) of reporter-positive cells over time as measured by flow cytometry after single transductions with GaLVenv retroviruses carrying the indicated latent EBV genes (n=6; three EBV-negative donors with two independent replicates each). Data correspond to medians with range. LMP1-expressing cells were positively selected approaching 100% of reporter-positive cells over time. GFP+, LMP2A+ and EBNA2+ cells were dying out from day 25 (indicated by a cross in each case). **(C–E)** LMP1/EBNA2-coexpressing cells are capable of long-term proliferative outgrowth, while LMP1/LMP2A-coexpressing cells are not. Plotted data correspond to medians with range. **(C, D)** Percentage of reporter-positive cell-subpopulations over time as measured by flow cytometry after double transductions with LMP1, and either EBNA2 or LMP2A. Left: Flow cytometry analysis of B cells from one representative donor identifies four subpopulations: double-transduced (mCherry and GFP double-positive), single transduced (either mCherry- or GFP-positive) and non-transduced (reporter-negative). Right: Change of the four cell subpopulations over time. **(C)** LMP1/EBNA2 double-transduction. Only LMP1/EBNA2-coexpressing cells showed significant outgrowth (paired, two-tailed t-test (day 60), *p* < 0.001 (***); n=15 (three EBV-negative donors with five independent replicates each). **(D)** LMP1/LMP2A double-transduction (paired, two-tailed t-test (day 60), *p* = 0.87 (ns = not significant); n=6 (three EBV-negative donors with two independent replicates each). **(E)** Fold changes of total living cells over time (irrespective of reporter-expression) (ordinary one-way ANOVA with Tukey’s multiple comparisons test (day 60), *p* = 0.02 (*), *p* < 0.001 (***); n=6 (three EBV-negative donors with two independent replicates each).

In order to pinpoint a minimum set of EBV latent genes required for human B cell immortalization, we cultured human peripheral B cells from three EBV-negative donors on CD40L/IL21-expressing feeder cells (33), and transduced them, individually or in combination, with GaLVenv retroviruses carrying LMP1, opEBNA2 or LMP2A (**Figure 2**). Feeder cells were withdrawn on day 3 after transduction, and cell growth was monitored by flow cytometric analysis of reporter expression or of the number of total cells in the presence of counting beads (**Figure 2A**). When transduced individually, LMP1-expressing cells survived, expanded until day 10, and then persisted without further expansion for the rest of the 60-day observation period, while EBNA2- and LMP2A-expressing cells steadily decreased in numbers, dying out by day 25 (**Figure 2B**). These results suggest that none of the three EBV latent genes is capable by itself of growth-transforming primary human B cells.

In a separate series of experiments, we co-transduced LMP1 either with EBNA2 or with LMP2A, and monitored the growth of the resulting four cellular subpopulations by their differential reporter expression in co-culture, in the same culture well (**Figures 2C, D**). LMP1/EBNA2-coexpressing cells overtook the cultures within 25 days, and outcompeted the cells expressing LMP1-only while EBNA2-only expressing and non-transduced cells died out by day 15 (**Figure 2C**). In contrast to this, co-transduction of LMP1 and LMP2A did not show outgrowth of LMP1/LMP2A-double-positive cells (**Figure 2D**). Rather, LMP1-expressing and LMP1/LMP2A-co-expressing cells coexisted over the entire culture period, reaching equal proportions by day 60. Non-transduced and LMP2A-only expressing cells were barely detectable in co-culture (**Figure 2D**). These results suggest that EBNA2 expression confers a growth advantage to LMP1-expressing B cells while LMP2A expression does not.

We also measured the total number of single- and double-transduced cells in independent parallel cultures of virally transduced B cells. As evident from the change in cell numbers relative to the number of cells on day 3 after transduction, LMP1/EBNA2-expressing cells showed continuous and sustained growth over five orders of magnitude within the 60-day observation period (**Figure 2E**). In contrast to this, cells expressing LMP1 in combination with LMP2A expanded only about 10-fold within the first 15 days, and then remained stable, similar to cells expressing LMP1 only (**Figure 2E**). These results confirmed that EBNA2, but not LMP2A, endowed LMP1-expressing B cells with the ability to grow continuously in culture.

On day 60 after transduction, stable ‘synthetic’ LMP1/EBNA2-coexpressing (synL1E2) cell lines had emerged which expressed LMP1 and EBNA2 proteins at comparable levels to LCLs that had been established in parallel from the same donors (**Supplementary Figure 2A**). However, in contrast to LCLs, synL1E2 lines did not express EBER1 and EBER2 RNAs, excluding the possibility that the enhanced growth potential of synL1E2 lines was caused by an unintentional EBV infection (**Supplementary Figure 2B**).

In order to confirm that synL1E2 lines were dependent on the continuous expression of LMP1 and EBNA2, we designed LMP1 and EBNA2-specific gRNAs using our CrispRGold algorithm (48) and performed gene editing by electroporation of gRNA/Cas9 ribonucleoprotein (RNP) complexes to introduce DNA double-strand breaks (49). Repair of these DNA lesions by non-homologous end joining generates insertions or deletions (Indels) that can disrupt gene expression by causing frameshift mutations that introduce premature stop codons, leading to gene knock-outs. In order to determine the proportions of alleles with frameshift (knock-out) mutations, we performed amplicon deep sequencing on growing cell populations that had undergone LMP1 or EBNA2 mutagenesis (**Supplementary Figure 3A**). As shown in **Supplementary Figure 3B**, when compared to day 3 post gene editing, the percentage of knock-out alleles was greatly reduced at the later time points. By day 10 (LMP1) and day 12 (EBNA2) cells carrying knock-out mutations in LMP1 and EBNA2 had largely disappeared from the edited cell populations, indicating that synL1E2 cell lines are addicted to continuous expression of both EBV latent genes, LMP1 and EBNA2.

Taken together, our results suggest that LMP1 and EBNA2 represent a minimal set of EBV latent genes sufficient to immortalize human primary B cells.

### LMP1/EBNA2-coexpressing synL1E2 cell lines show similarities and differences to EBV B95-8 transformed LCLs

**Figure 3:**
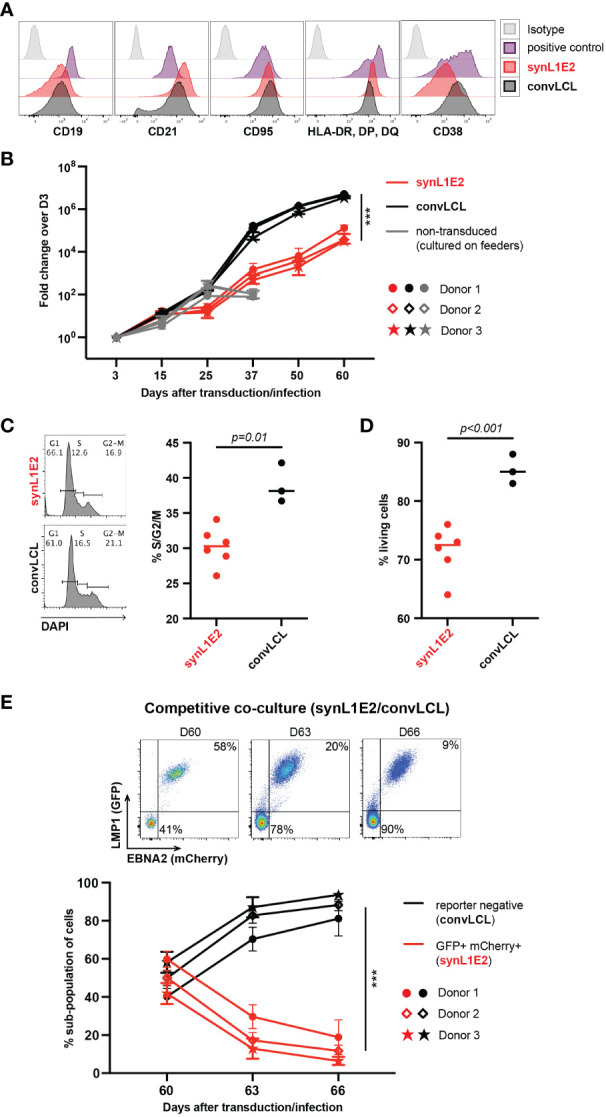
LMP1/EBNA2-coexpressing B cells are phenotypically similar to conventional lymphoblastoid cell lines (convLCLs), but have a more restricted proliferative capacity. **(A)** Activation and B cell linage marker profiles of human B cells 60 days after transduction with LMP1 and EBNA2 (synL1E2) or infection with EBV wildtype strain B95-8 (convLCLs). SynL1E2 cells display similar, but not identical B cell activation marker profiles as convLCLs (see also **Supplementary Figure 4A**). Antibodies with matching isoptypes were used as negative controls. **(B)** LMP1/EBNA2-coexpressing synL1E2 lines (red) expanded more slowly than convLCLs (black). B cells were cultured and treated as shown in **Figure 2A**, or infected on day 0. As negative control for this experiment, activated, non-transduced B cells were cultured on CD40L/IL-21-expressing feeder cells which were replenished every 3 to 4 days (grey curves; from day 50 on living cells were not detectable). Plotted data represent median with range for three negative donors with five independent replicates each. Unpaired, two-tailed t-test with Welch’s correction (day 60), *p* < 0.001 (***) (n=15 for all three donors combined). **(C, D)** SynL1E2 cultures contain less actively dividing and living cells compared to convLCLs (day 80 after double-transduction in discontinous culture: 60 days culture plus 20 days re-culture after freezing and thawing). Each dot represents one independent cell line, bars indicate median. **(C)** DNA content analysis by flow cytometry. Left panel: representative result for a synL1E2 and a convLCL line with gating strategy for G1, S and G2/M phase. Right panel: Quantification of dividing cells in S/G2/M phase combined. Unpaired, two-tailed t-test with Welch’s correction, *p* = 0.01. **(D)** Live cell count by trypan blue exclusion in synL1E2 cultures. Unpaired, two-tailed t-test with Welch’s correction, *p* < 0.001. **(E)** Outgrowth of convLCLs (black) over synL1E2 cell lines (red) in competitive co-culture. SynL1E2 cell lines and convLCLs (on day 60 after transduction or infection, respectively) were co-cultured, and their growth was monitored by flow cytometry. Upper panels: Flow cytometry of co-cultured B cell lines identifies double-transduced synL1E2 cells (mCherry and GFP double-positive) and convLCLs (reporter-negative) at the indicated time points. ConvLCLs overtook the cultures after three days. Lower Panel: Change of the subpopulations over time. Plotted data correspond to means with range for three EBV-negative donors with two independent replicates each. Paired, two-tailed t-test (day 66), *p* < 0.001 (***) (n=6 for all three donors combined).

In order to characterize LMP1/EBNA2-expressing synL1E2 B cell lines, we directly compared them to LCLs that we generated by infection of human primary B cells pre-activated on CD40L/IL-21-expressing feeder cells with the prototypic EBV wildtype strain B95-8 (38, 39) (convLCLs). Both, synL1E2 cell lines and convLCLs were positive for the B cell markers CD19 and CD20, and expressed high levels of CD21, CD95 (Fas), HLA-II, CD23 and CD54 (ICAM-1) (**Figure 3A**; **Supplementary Figure 4A**), in line with the ability of LMP1 and EBNA2 to induce B cell activation markers (50–54) and previous characterizations of LCLs as highly activated B cells (1, 55). It should be noted, however, that some surface markers were differentially expressed between transduced and infected cells. The reasons for this discrepancy remain unclear, and might be due to slightly different expression levels of the viral proteins, or lack of the complete EBV latent gene expression program in transduced cells, or different origins of transduced and infected cells.

Consistent with their highly activated state, synL1E2 lines and convLCLs proliferated steadily, although synL1E2 lines proliferated less vigorously than convLCLs. This was due on the one hand to a lower proportion of actively dividing cells, and on the other hand to a lower proportion of living cells in the culture (**Figures 3B–D**). When synL1E2s were placed in competitive co-culture with convLCLs on day 60 after retroviral transduction and EBV infection, respectively, synL1E2s were quickly outcompeted by convLCLs (**Figure 3E**). Likewise, newly EBV-infected primary B cells quickly overtook LMP1/EBNA2-coexpressing B cells upon co-culture 15 days after infection and transduction, respectively (**Supplementary Figure 4B**). These results indicate that the slower growth of synL1E2 cell lines is cell-intrinsic.

Conditionally activated EBNA2 suppresses IgM transcription and surface IgM expression in LCLs (56). We observed that LMP1/EBNA2 coexpressing B cells rapidly lost surface IgM expression after retroviral transduction, to a greater extent than LCLs, (**Supplementary Figure 5A**), but retained the ability to secrete antibody, with five out of six synL1E2 lines secreting IgM only, in contrast to some of the convLCLs which additionally secreted IgG and IgA (**Supplementary Figure 5B**). Sequence analysis of the productively rearranged immunoglobulin heavy chain loci revealed that both, synL1E2 lines and convLCLs were oligoclonal. The heavy chain V genes of synL1E2 lines were unmutated (with one exception), whereas the productively rearranged VH genes in convLCLs carried varying numbers of somatic mutations (**Supplementary Figure 5C**), in agreement with earlier studies (57).

Taken together, these results suggest that synL1E2 cell lines may be derived predominantly from naïve B cells, while our convLCLs represent a broader spectrum of B cell subsets including memory B cells. The phenotype of our synL1E2 lines and convLCLs was in line with previous characterization of LCLs as activated B cells or plasmablasts (55).

### LMP1/EBNA2-coexpressing B cells show tumorigenic growth *in vivo*

**Figure 4:**
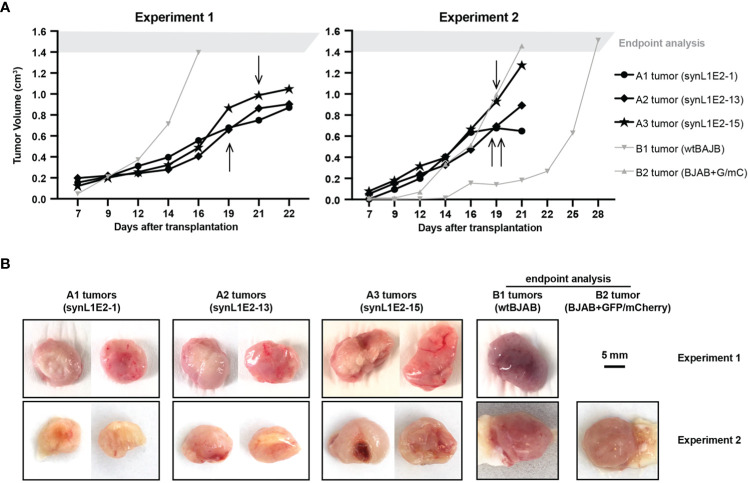
LMP1/EBNA2-coexpressing synL1E2 cells grow *in vivo* in severely immunodeficient mice. **(A)** Selected synL1E2 B cell lines were transplanted subcutaneously into NOD/SCID/γcnull (NOG) mice. Malignant BJAB cells were used as reference. The volume of the tumor nodules at the transplant site was monitored over time. Arrows indicate the time point at which skin ulcerations appeared in five out of six synL1E2 recipient hosts; mice had to be sacrificed shortly after (humane endpoint). Experiment 1 and 2 were independent of each other. **(B)** Pictures of dissected tumors at the time points at which mice had to be sacrificed, corresponding to the experiments shown in panel **(A)**. A1 to A3 tumors, synL1E2 tumors; B1 and B2 tumors, BJAB tumors. Sizebar, 5 mm.

To evaluate the growth potential of LMP1/EBNA2-coexpressing B cells *in vivo*, three independent synL1E2 cell lines were transplanted into severely immunodeficient NOD/SCID/γcnull (NOG) mice that lack mature T, B and NK cells, and have dysfunctional macrophages and dendritic cells, as well as a reduced complement activity (58). All synL1E2 cell lines developed tumor-like nodules at the injection sites. Five out of six mice carrying synL1E2 tumors had to be sacrificed due to skin ulcerations at the injection site before the tumors reached the predefined endpoint of 1.5 cm^3^ tumor volume (**Figure 4A**). The dissected synL1E2 tumors were similar in size to BJAB reference tumors which had reached the endpoint (**Figure 4B**). Immunofluorescence and PCR analysis revealed that the synL1E2 tumors harbored a majority of EBNA2-positive, human cells, as well as a smaller number of infiltrating, CD45-positive mouse cells (**Supplementary Figures 6A–E**). SynL1E2 tumors contained actively dividing cells as identified by staining with a human-specific anti-Ki-67 antibody (**Supplementary Figure 7A**). The infiltrating mouse cells corresponded mostly to CD11b/Ly6G-positive neutrophils, possibly attracted by the various pro-inflammatory cytokines and chemokines secreted by synL1E2 cell lines, including IL-8 which is known to cross-react with mouse IL-8 receptor (**Supplementary Figures 7B, C**) (59).

Taken together, our results suggest that the LMP1/EBNA2-coexpressing cells are capable of tumorous growth *in vivo*.

## Discussion

Cell lines generated by EBV-driven B cell immortalization in cell culture serve as a model for EBV-transformed B cells arising in infectious mononucleosis, PLD or under immunosuppression. The transformation process can be divided into three phases, a pre-latent phase (up to 8 days post infection (p. i.)), the outgrowth of lymphoblastoid cells (up to 4 weeks p. i.) and the growth of established LCLs (16). Despite extensive research over decades, it has remained difficult to define the exact functions and contributions of individual EBV latent genes to B cell immortalization (16). Oncogenic EBV latent proteins have been compared to Swiss army knives, each one exerting many different, as well as partially overlapping functions (7).

Here, we approached this problem by asking whether individual EBV genes are able to transform primary human B cells. We used a gain-of-function approach by retrovirally overexpressing selected EBV latent proteins, and assessing the growth behavior of the transduced B cells. We focused on LMP1 and EBNA2, because of their importance for B cell immortalization in culture (16) and because of the following three observations from our previous work: (i) Transgenic expression of LMP1 in mice causes spontaneous monoclonal B cell lymphomas (31); (ii) some of these B cell lymphomas had acquired mutations in *Ebf1* as a secondary oncogenic event (32); and (iii) co-expression of LMP1 and cellular EBF1 is able to recapitulate EBV-mediated transformation in mice, and to promote survival and proliferation of human tonsillar B cells in culture (32). Since EBF1 is a cellular effector of EBNA2, that is necessary for full induction of oncogenic MYC levels by EBNA2 in the context of EBV-mediated B cell transformation (20), a logical step was to ask, whether LMP1 and EBNA2 together can transform human B cells. Based on our results, we make the following three main points:

First, optimization of the EBNA2 CDS at the transcriptional and translational level overcame an inability to express EBNA2 in primary mouse and human B cells at a level that would allow the study of EBNA2’s growth-modifying properties ( (32) and this study). Optimized EBNA2 could be expressed at a protein level comparable to that in LCLs, but was not sufficient to sustain the growth and proliferation of EBNA2-expressing primary B cells beyond day 6 (see **Figure 2B**). This differed from the behavior of primary B cells expressing LMP1, which proliferated for nearly two weeks, before plateauing for the rest of the 60-day observation period (**Figures 2B, E**).

Second, co-expression of only two EBV latent genes, EBNA2 and LMP1, proved to be sufficient for the growth transformation of activated human primary B cells in culture. LMP1/EBNA2 co-expressing cells behaved similarly to LMP1-only transduced cells for about two weeks, but then continued to proliferate and grew out as stable cell lines (**Figure 2E**). Our experiments bypass the initial, critical step of reprogramming resting B cells (the pre-latent phase), because we used activated, proliferating B cells for transduction. In EBV infection in cell culture, with resting B cells as target cells, EBV latent genes act in a strict, temporally controlled manner: EBNA2 is expressed first, while LMP1 becomes expressed around day 4 and reaches full LCL expression levels only three weeks after infection (25, 55). In such a context EBNA3C is essential for the outgrowth of lymphoblastoid cells as well as the growth of established LCLs by regulating host genes protective of cell death and by suppressing plasma cell differentiation (16, 60–63).

By contrast, in our experiments, LMP1 is always co-expressed with EBNA2 at high levels, which may substitute to some extent for functions of other EBV latent proteins, in particular the pro-survival functions mediated by EBNA3C. In this context, it is interesting to note that synL1E2 cell cultures proliferated less strongly and contained more dead cells than established LCLs which express the full complement of EBV latent genes (**Figures 3B, D**).

Third, synL1E2 B cell lines established from LMP1/EBNA2 co-expressing cells appeared truly growth-transformed, because they showed consistent tumorous growth in a xenotransplantation model (**Figure 4B**).

Limitations: We did not investigate the mechanism of LMP1 and EBNA2 cooperation. The possibility of cooperative induction of oncogenic MYC levels (as outlined in the Introduction) could be addressed by constructing EBNA2 deletion mutants defective for recruitment of the cellular effectors EBF1 and RBPJ (20, 64). We tried, but were unable to express EBNA3 proteins at sufficient levels in primary human B cells, a problem that might be addressed in the future by synthesizing expression-optimized coding regions (37). We did not attempt to express other EBV latent genes. Moreover, we did not try to express LMP2A together with EBNA2. Indeed, LMP2A can replace BCR signaling in B cell development by providing pro-survival signals (65). However, primary human B cells transduced with LMP2A or EBNA2 alone showed a growth deficit when compared to GFP reporter-positive control cells which were dying out by day 37 (**Figure 2B**). Furthermore, we could not directly compare *in vivo* tumorous growth of LCLs with synL1E2 cell lines in the xenotransplantation model, because of regulatory restrictions regarding *in vivo* mouse work with cell lines that shed intact EBV. Despite these limitations, our results show that LMP1 and EBNA2 constitute a minimal set of EBV proteins sufficient for B cell transformation.

## Data availability statement

The original contributions presented in the study are included in the article/supplementary material. Further inquiries can be directed to the corresponding author.

## Ethics statement

The studies involving humans were approved by Institutional Review Board of the University of Cologne and respective local Institutional Review Boards (study protocol 16-054). The studies were conducted in accordance with the local legislation and institutional requirements. The participants provided their written informed consent to participate in this study. The animal study was approved by Landesamt für Gesundheit und Soziales Berlin (Reg 0010/19). The study was conducted in accordance with the local legislation and institutional requirements.

## Author contributions

JZ: Investigation, Visualization, Writing – review & editing, Formal analysis. TS: Writing – review & editing. XL: Writing – review & editing. LG: Writing – review & editing, Resources. KdlR: Writing – review & editing, Resources. MS: Investigation, Writing – review & editing, Resources. FK: Writing – review & editing, Resources. CK: Visualization, Writing – original draft, Writing – review & editing. KR: Conceptualization, Funding acquisition, Supervision, Writing – review & editing, Visualization.

## References

[1] Young LS Arrand JR Murray PG. Chapter 27 EBV gene expression and regulation. In: Arvin A Campadelli-Fiume G Mocarski E Moore PS Roizman B Whitley R Yamanishi K, editors. Human herpesviruses: biology, therapy, and immunoprophylaxis. Cambridge: Cambridge University Press (2007). Available at: https://www.ncbi.nlm.nih.gov/books/NBK47431/. 21348071

[2] Hjalgrim H Friborg J Melbye M. Chapter 53 The epidemiology of EBV and its association with Malignant disease. In: Arvin A Campadelli-Fiume G Mocarski E Moore PS Roizman B Whitley R Yamanashi K, editors. Human herpesviruses: biology, therapy, and immunoprophylaxis. Cambridge: Cambridge University Press (2007). Available at: https://www.ncbi.nlm.nih.gov/books/NBK47424/. 21348109

[3] Mentzer AJ Brenner N Allen N Littlejohns TJ Chong AY Cortes A. Identification of host–pathogen-disease relationships using a scalable multiplex serology platform in UK Biobank. Nat Commun (2022) 13:1818. doi: 10.1038/s41467-022-29307-3 35383168 PMC8983701

[4] Taylor GS Long HM Brooks JM Rickinson AB Hislop AD. The immunology of Epstein-Barr virus–induced disease. Annu Rev Immunol (2015) 33:1–35. doi: 10.1146/annurev-immunol-032414-112326 25706097

[5] Hislop AD Taylor GS Sauce D Rickinson AB. Cellular responses to viral infection in humans: lessons from Epstein-Barr virus. Annu Rev Immunol (2007) 25:587–617. doi: 10.1146/annurev.immunol.25.022106.141553 17378764

[6] Münz C. Epstein–barr virus-specific immune control by innate lymphocytes. Front Immunol (2017) 8:1658. doi: 10.3389/fimmu.2017.01658 29225606 PMC5705607

[7] Münz C. Latency and lytic replication in Epstein–Barr virus-associated oncogenesis. Nat Rev Microbiol (2019) 17:691–700. doi: 10.1038/s41579-019-0249-7 31477887

[8] Bjornevik K Cortese M Healy BC Kuhle J Mina MJ Leng Y. Longitudinal analysis reveals high prevalence of Epstein-Barr virus associated with multiple sclerosis. Science (2022) 375:296–301. doi: 10.1126/science.abj8222 35025605

[9] Pender MP Csurhes PA Burrows JM Burrows SR. Defective T-cell control of Epstein–Barr virus infection in multiple sclerosis. Clin Transl Immunol (2017) 6:e126. doi: 10.1038/cti.2016.87 PMC5292561 28197337

[10] Cohen JI Fauci AS Varmus H Nabel GJ. Epstein-barr virus: an important vaccine target for cancer prevention. Sci Transl Med (2011) 3:107fs7. doi: 10.1126/scitranslmed.3002878 PMC3501269 22049067

[11] Shannon-Lowe C Rickinson A. The global landscape of EBV-associated tumors. Front Oncol (2019) 9:713. doi: 10.3389/fonc.2019.00713 31448229 PMC6691157

[12] Wong Y Meehan MT Burrows SR Doolan DL Miles JJ. Estimating the global burden of Epstein–Barr virus-related cancers. J Cancer Res Clin Oncol (2022) 148:31–46. doi: 10.1007/s00432-021-03824-y 34705104 PMC8752571

[13] Young LS Rickinson AB. Epstein–Barr virus: 40 years on. Nat Rev Cancer (2004) 4:757–68. doi: 10.1038/nrc1452 15510157

[14] Thorley-Lawson DA. EBV persistence - introducing the virus. Curr Top Microbiol Immunol (2015) 390:151–209. doi: 10.1007/978-3-319-22822-8_8 26424647 PMC5125397

[15] Babcock GJ Hochberg D Thorley-Lawson DA. The expression pattern of Epstein-Barr virus latent genes *in vivo* is dependent upon the differentiation stage of the infected B cell. Immunity (2000) 13:497–506. doi: 10.1016/s1074-7613(00)00049-2 11070168

[16] Pich D Mrozek-Gorska P Bouvet M Sugimoto A Akidil E Grundhoff A. First days in the life of naive human B lymphocytes infected with Epstein-Barr virus. mBio (2019) 10:e01723–19. doi: 10.1128/mbio.01723-19 PMC6751056 31530670

[17] Hammerschmidt W Sugden B. Genetic analysis of immortalizing functions of Epstein–Barr virus in human B lymphocytes. Nature (1989) 340:393–7. doi: 10.1038/340393a0 2547164

[18] Cohen JI Wang F Mannick J Kieff E. Epstein-Barr virus nuclear protein 2 is a key determinant of lymphocyte transformation. Proc Natl Acad Sci (1989) 86:9558–62. doi: 10.1073/pnas.86.23.9558 PMC298536 2556717

[19] Kempkes B Ling PD. EBNA2 and its coactivator EBNA-LP. Curr Top Microbiol Immunol (2015) 391:35–59. doi: 10.1007/978-3-319-22834-1_2 26428371

[20] Beer S Wange LE Zhang X Kuklik-Roos C Enard W Hammerschmidt W. EBNA2-EBF1 complexes promote MYC expression and metabolic processes driving S-phase progression of Epstein-Barr virus–infected B cells. Proc Natl Acad Sci (2022) 119:e2200512119. doi: 10.1073/pnas.2200512119 35857872 PMC9335265

[21] Zimber-Strobl U Strobl LJ. EBNA2 and Notch signalling in Epstein–Barr virus mediated immortalization of B lymphocytes. Semin Cancer Biol (2001) 11:423–34. doi: 10.1006/scbi.2001.0409 11669604

[22] Kaiser C Laux G Eick D Jochner N Bornkamm GW Kempkes B. The Proto-Oncogene c- myc Is a Direct Target Gene of Epstein-Barr Virus Nuclear Antigen 2. J Virol (1999) 73:4481–4. doi: 10.1128/jvi.73.5.4481-4484.1999 PMC104340 10196351

[23] Glaser LV Rieger S Thumann S Beer S Kuklik-Roos C Martin DE. EBF1 binds to EBNA2 and promotes the assembly of EBNA2 chromatin complexes in B cells. PloS Pathog (2017) 13:e1006664. doi: 10.1371/journal.ppat.1006664 28968461 PMC5638620

[24] Zolotarev N Bayer M Grosschedl R. EBF1 is continuously required for stabilizing local chromatin accessibility in pro-B cells. Proc Natl Acad Sci (2022) 119:e2210595119. doi: 10.1073/pnas.2210595119 36409886 PMC9860308

[25] Kieser A Sterz KR. The latent membrane protein 1 (LMP1). Curr Top Microbiol Immunol (2015) 391:119–49. doi: 10.1007/978-3-319-22834-1_4 26428373

[26] Baumforth KR Young LS Flavell KJ Constandinou C Murray PG. The Epstein-Barr virus and its association with human cancers. Mol Pathol (1999) 52:307. doi: 10.1136/mp.52.6.307 10748864 PMC395716

[27] Soni V Cahir-McFarland E Kieff E. LMP1 TRAFficking activates growth and survival pathways. Adv Exp Med Biol (2007) 597:173–87. doi: 10.1007/978-0-387-70630-6_14 17633026

[28] Rastelli J Hömig-Hölzel C Seagal J Müller W Hermann AC Rajewsky K. LMP1 signaling can replace CD40 signaling in B cells in *vivo* and has unique features of inducing class-switch recombination to IgG1. Blood (2008) 111:1448–55. doi: 10.1182/blood-2007-10-117655 18006702

[29] Pratt ZL Zhang J Sugden B. The latent membrane protein 1 (LMP1) oncogene of Epstein-Barr virus can simultaneously induce and inhibit apoptosis in B cells. J Virol (2012) 86:4380–93. doi: 10.1128/jvi.06966-11 PMC3318665 22318153

[30] Voigt S Sterz KR Giehler F Mohr A-W Wilson JB Moosmann A. A central role of IKK2 and TPL2 in JNK activation and viral B-cell transformation. Nat Commun (2020) 11:685. doi: 10.1038/s41467-020-14502-x 32019925 PMC7000802

[31] Zhang B Kracker S Yasuda T Casola S Vanneman M Hömig-Hölzel C. Immune surveillance and therapy of lymphomas driven by Epstein-Barr virus protein LMP1 in a mouse model. Cell (2012) 148:739–51. doi: 10.1016/j.cell.2011.12.031 PMC3313622 22341446

[32] Sommermann T Yasuda T Ronen J Wirtz T Weber T Sack U. Functional interplay of Epstein-Barr virus oncoproteins in a mouse model of B cell lymphomagenesis. Proc Natl Acad Sci (2020) 117:14421–32. doi: 10.1073/pnas.1921139117 PMC7322082 32522871

[33] Caeser R Re MD Krupka JA Gao J Lara-Chica M Dias JML. Genetic modification of primary human B cells to model high-grade lymphoma. Nat Commun (2019) 10:4543. doi: 10.1038/s41467-019-12494-x 31586074 PMC6778131

[34] Högstrand K Grandien A. MYC-driven Malignant transformation of mature murine B cells requires inhibition of both intrinsic apoptosis and p53 activity. Eur J Immunol (2019) 49:375–85. doi: 10.1002/eji.201847585 30281155

[35] Dirmeier U Hoffmann R Kilger E Schultheiss U Briseño C Gires O. Latent membrane protein 1 of Epstein–Barr virus coordinately regulates proliferation with control of apoptosis. Oncogene (2005) 24:1711–7. doi: 10.1038/sj.onc.1208367 15674340

[36] Naito M Hainz U Burkhardt UE Fu B Ahove D Stevenson KE. CD40L-Tri, a novel formulation of recombinant human CD40L that effectively activates B cells. Cancer Immunol Immunother (2013) 62:347–57. doi: 10.1007/s00262-012-1331-4 PMC3569584 22926059

[37] Fath S Bauer AP Liss M Spriestersbach A Maertens B Hahn P. Multiparameter RNA and codon optimization: A standardized tool to assess and enhance autologous mammalian gene expression. PloS One (2011) 6:e17596. doi: 10.1371/journal.pone.0017596 21408612 PMC3048298

[38] Raab-Traub N Dambaugh T Kieff E. DNA of Epstein-Barr virus VIII: B95-8, the previous prototype, is an unusual deletion derivative. Cell (1980) 22:257–67. doi: 10.1016/0092-8674(80)90173-7 6253079

[39] Ba Abdullah MM Palermo RD Palser AL Grayson NE Kellam P Correia S. Heterogeneity of the Epstein-Barr virus (EBV) major internal repeat reveals evolutionary mechanisms of EBV and a functional defect in the prototype EBV strain B95-8. J Virol (2017) 91:e00920-17. doi: 10.1128/jvi.00920-17 28904201 PMC5686732

[40] Janssens W Chuah MKL Naldini L Follenzi A Collen D Saint-Remy J-M. Efficiency of onco-retroviral and lentiviral gene transfer into primary mouse and human B-lymphocytes is pseudotype dependent. Hum Gene Ther (2003) 14:263–76. doi: 10.1089/10430340360535814 12639306

[41] Serafini M Naldini L Introna M. Molecular evidence of inefficient transduction of proliferating human B lymphocytes by VSV-pseudotyped HIV-1-derived lentivectors. Virology (2004) 325:413–24. doi: 10.1016/j.virol.2004.04.038 15246279

[42] Mock U Thiele R Uhde A Fehse B Horn S. Efficient lentiviral transduction and transgene expression in primary human B cells. Hum Gene Ther Methods (2012) 23:408–15. doi: 10.1089/hgtb.2012.160 23240650

[43] Lynch DT Zimmerman JS Rowe DT. Epstein–Barr virus latent membrane protein 2B (LMP2B) co-localizes with LMP2A in perinuclear regions in transiently transfected cells. J Gen Virol (2002) 83:1025–35. doi: 10.1099/0022-1317-83-5-1025 11961256

[44] Lam N Sugden B. LMP1, a viral relative of the TNF receptor family, signals principally from intracellular compartments. EMBO J (2003) 22:3027–38. doi: 10.1093/emboj/cdg284 PMC162136 12805217

[45] Verweij FJ van EMAJ ES H Vendrig T Wurdinger T Cahir-McFarland E. LMP1 association with CD63 in endosomes and secretion via exosomes limits constitutive NF-κB activation. EMBO J (2011) 30:2115–29. doi: 10.1038/emboj.2011.123 PMC3117644 21527913

[46] Peng Q Wang L Qin Z Wang J Zheng X Wei L. Phase separation of Epstein-Barr virus EBNA2 and its coactivator EBNALP controls gene expression. J Virol (2020) 94:e01771–19. doi: 10.1128/jvi.01771-19 PMC7081900 31941785

[47] Yang Y Ye X Dai R Li Z Zhang Y Xue W. Phase separation of Epstein-Barr virus EBNA2 protein reorganizes chromatin topology for epigenetic regulation. Commun Biol (2021) 4:967. doi: 10.1038/s42003-021-02501-7 34400762 PMC8368186

[48] Graf R Li X Chu VT Rajewsky K. sgRNA sequence motifs blocking efficient CRISPR/cas9-mediated gene editing. Cell Rep (2019) 26:1098–1103.e3. doi: 10.1016/j.celrep.2019.01.024 30699341 PMC6352712

[49] Wu C-AM Roth TL Baglaenko Y Ferri DM Brauer P Zuniga-Pflucker JC. Genetic engineering in primary human B cells with CRISPR-Cas9 ribonucleoproteins. J Immunol Methods (2018) 457:33–40. doi: 10.1016/j.jim.2018.03.009 29614266 PMC6124898

[50] Wang F Gregory CD Rowe M Rickinson AB Wang D Birkenbach M. Epstein-Barr virus nuclear antigen 2 specifically induces expression of the B-cell activation antigen CD23. Proc Natl Acad Sci (1987) 84:3452–6. doi: 10.1073/pnas.84.10.3452 PMC304889 3033649

[51] Calender A Billaud M Aubry JP Banchereau J Vuillaume M Lenoir GM. Epstein-Barr virus (EBV) induces expression of B-cell activation markers on in *vitro* infection of EBV-negative B-lymphoma cells. Proc Natl Acad Sci (1987) 84:8060–4. doi: 10.1073/pnas.84.22.8060 PMC299477 2825176

[52] Cordier M Calender A Billaud M Zimber U Rousselet G Pavlish O. Stable transfection of Epstein-Barr virus (EBV) nuclear antigen 2 in lymphoma cells containing the EBV P3HR1 genome induces expression of B-cell activation molecules CD21 and CD23. J Virol (1990) 64:1002–13. doi: 10.1128/jvi.64.3.1002-1013.1990 PMC249210 2154588

[53] Uchida J Yasui T Takaoka-Shichijo Y Muraoka M Kulwichit W Raab-Traub N. Mimicry of CD40 signals by Epstein-Barr virus LMP1 in B lymphocyte responses. Science (1999) 286:300–3. doi: 10.1126/science.286.5438.300 10514374

[54] Le Clorennec C Youlyouz-Marfak I Adriaenssens E Coll J Bornkamm GW Feuillard J. EBV latency III immortalization program sensitizes B cells to induction of CD95-mediated apoptosis via LMP1: role of NF- B, STAT1, and p53. Blood (2006) 107:2070–8. doi: 10.1182/blood-2005-05-2053 16317104

[55] Mrozek-Gorska P Buschle A Pich D Schwarzmayr T Fechtner R Scialdone A. Epstein–Barr virus reprograms human B lymphocytes immediately in the prelatent phase of infection. Proc Natl Acad Sci (2019) 116:16046–55. doi: 10.1073/pnas.1901314116 PMC6690029 31341086

[56] Jochner N Eick D Zimber-Strobl U Pawlita M Bornkamm GW Kempkes B. Epstein-Barr virus nuclear antigen 2 is a transcriptional suppressor of the immunoglobulin mu gene: implications for the expression of the translocated c-myc gene in Burkitt’s lymphoma cells. EMBO J (1996) 15:375–82. doi: 10.1002/j.1460-2075.1996.tb00367.x PMC449952 8617212

[57] Heath E Begue-Pastor N Chaganti S Croom-Carter D Shannon-Lowe C Kube D. Epstein-barr virus infection of naïve B cells *in vitro* frequently selects clones with mutated immunoglobulin genotypes: implications for virus biology. PloS Pathog (2012) 8:e1002697. doi: 10.1371/journal.ppat.1002697 22589726 PMC3349760

[58] Ito M Hiramatsu H Kobayashi K Suzue K Kawahata M Hioki K. NOD/SCID/γcnull mouse: an excellent recipient mouse model for engraftment of human cells. Blood (2002) 100:3175–82. doi: 10.1182/blood-2001-12-0207 12384415

[59] Fan X Patera AC Pong-Kennedy A Deno G Gonsiorek W Manfra DJ. Murine CXCR1 is a functional receptor for GCP-2/CXCL6 and interleukin-8/CXCL8*. J Biol Chem (2007) 282:11658–66. doi: 10.1074/jbc.m607705200 17197447

[60] Tomkinson B Robertson E Kieff E. Epstein-Barr virus nuclear proteins EBNA-3A and EBNA-3C are essential for B-lymphocyte growth transformation. J Virol (1993) 67:2014–25. doi: 10.1128/jvi.67.4.2014-2025.1993 PMC240270 8445720

[61] Maruo S Wu Y Ishikawa S Kanda T Iwakiri D Takada K. Epstein–Barr virus nuclear protein EBNA3C is required for cell cycle progression and growth maintenance of lymphoblastoid cells. Proc Natl Acad Sci (2006) 103:19500–5. doi: 10.1073/pnas.0604919104 PMC1748255 17159137

[62] Ohashi M Hayes M McChesney K Johannsen E. Epstein-Barr virus nuclear antigen 3C (EBNA3C) interacts with the metabolism sensing C-terminal binding protein (CtBP) repressor to upregulate host genes. PloS Pathog (2021) 17:e1009419. doi: 10.1371/journal.ppat.1009419 33720992 PMC7993866

[63] Skalska L White RE Franz M Ruhmann M Allday MJ. Epigenetic repression of p16INK4A by latent Epstein-Barr virus requires the interaction of EBNA3A and EBNA3C with ctbp. PloS Pathog (2010) 6:e1000951. doi: 10.1371/journal.ppat.1000951 20548956 PMC2883600

[64] Ling PD Hayward SD. Contribution of conserved amino acids in mediating the interaction between EBNA2 and CBF1/RBPJk. J Virol (1995) 69:1944–50. doi: 10.1128/jvi.69.3.1944-1950.1995 PMC188813 7853539

[65] Casola S Otipoby KL Alimzhanov M Humme S Uyttersprot N Kutok JL. B cell receptor signal strength determines B cell fate. Nat Immunol (2004) 5:317–27. doi: 10.1038/ni1036 14758357

