# Supplementary material for: LMP1 and EBNA2 constitute a minimal set of EBV genes for transformation of human B cells
Source: Front Immunol. 2023 Dec 19;14:1331730. doi: 10.3389/fimmu.2023.1331730 (PMC10758421; doi:10.3389/fimmu.2023.1331730)
Supplement: Supplementary file 1 [file DataSheet_1.pdf]

Supplementary Material

LMP1 and EBNA2 constitute a minimal set of EBV genes for transformation of human B cells

Jingwei Zhang, Thomas Sommermann, Xun Li, Lutz Gieselmann, Kathrin de la Rosa, Maria Stecklum, Florian Klein, Christine Kocks, Klaus Rajewsky

This PDF file has 25 pages and includes:

Extended/Supplementary Materials and Methods

Supplementary Table 1 to 3 with Legends

Supplementary References

Supplementary Figure 1 to 7 with Legends

Materials and Methods

Isolation and culture of B cells

Human PBMCs with unknown status of HIV, HBV, HCV or EBV infection ("untested") were collected by the blood bank at Charité University Hospital, Berlin, Germany (**Figure 1 and Supplementary Figure 1**; short-term *in vitro* cultures). Human PBMCs from three healthy donors that were tested negative for HIV, HBV, HCV and EBV (antigen, IgG and IgM) ("tested") were kindly provided by Dr. Florian Klein, Institute of Virology, University Hospital of Cologne, Germany (from **Figure 2** on and **Supplementary Figure 2** on; all long-term *in vitro* cultures). CD19+ B cells were isolated by positive selection using CD19 Microbeads (Miltenyi, Cat. No. 130-050-301), except for the experiment in **Supplementary Figure 1B**, in which a memory B cell isolation kit was used (Miltenyi, Cat. No. 130-093-546), and seeded at concentration of 0.5 million/ml, and cultured in RPMI-1640 medium (Gibco, Cat. No. 31870-025) with 20% FCS (supplemented with 102mM β-mercaptoethanol, Penicillin/Streptomycin and 10 μg/ml gentamycin). Human primary B cells were cultured under activating conditions either in the presence of 2 μg/ml recombinant human CD40L trimers (CD40L-Tri; purified in-house (34) and 100 ng/ml recombinant human IL-21 (Biozol, Cat. No. BLD-571208) or on follicular dendritic cell (FDC)-like feeder cells (cell line YK6-CD40Lg-IL21 corresponding to YK6 cells stably expressing CD40L and IL21) (31). YK6-CD40Lg-IL21 cells were cultured in RPMI-1640 medium with 10% FCS, gamma-irradiated in suspension at concentration of 5 million cells/ml in FCS (30-35 Gy), aliquoted and stored at -80°C. For stimulation of human primary B cells, an aliquot of feeder cells was thawed and seeded (one day before or on the same day of B cell isolation) to reach 80% confluency. All cells were cultured at 37°C and 5% CO2 in a humidified incubator. Every 4-5 days the activation stimulus was replenished (fresh medium with CD40L-Tri/IL-21 or irradiated YK6-CD40Lg-IL21 feeder cells).

MSCV retroviral transfer constructs

The coding region (CDS) of EBV strain B95-8 latent proteins EBNA2, LMP1 and LPMP2A was inserted into an MSCV backbone based on pMIG (Addgene plasmid #9044, alternative name MSCV IRES GFP) containing a fluorescent reporter (GFP, mCherry, or BFP) preceded by an internal ribosomal entry site (IRES). First, GatewayTM entry vectors (pENTR/D-TOPO) were generated. Then each CDS was transferred to MSCV-based destination vectors (containing attR elements and ccdb gene) with the desired reporter. The CDS of EBNA2 was optimized for protein expression in mammalian cells on the transcriptional, mRNA and translational level **(Supplementary Table 1**) and custom synthesized (GeneOptimizer Software, GeneArt Gene Synthesis Services, ThermoFisher) (35).

Transfection of 293T cells

HEK 293T/17 cells (ATTC CRL-1168) were seeded 1 day before transfection, to reach 80% confluency on the day of transfection. For a 10 cm petridish of cells, 10 μg of MSCV retroviral transfer plasmid was mixed with 25 μg polyethylenimine (PEI linear, Polysciences Europe, Cat. No. 23966-1) in 1 ml Opti-MEM (Reduced Serum Medium, Gibco, Cat. No. 31985-047). The mixture was vortexed for 10 seconds incubated at room temperature for 10 minutes, then added drop-wise to the cell culture dish. The cell culture medium was changed 24 hours after transfection, and 293T cells were trypsinized and analyzed by FACS 48 hours after transduction.

Retrovirus production and concentration

HEK 293T/17 packaging cells were transfected with retroviral packaging vectors pHIT60 (gag-pol) and pGaLVenv (envelope, construct GALV WT) (31), and MSCV-retroviral transfer plasmids, as described above. For lentivirus production, we used the packaging plasmids psPAX2 (gag-pol, Addgene plasmid #12260) and pMD2.G (VSV-G envelope, Addgene plasmid #12259) and the transfer plasmid pLV-mCherry (Addgene plasmid #36084). Virus-containing cell culture supernatant was collected 2 or 3 days after transfection, filtered (0.45 µm, PES), and GaLVenv pseudotyped retroviral particles were enriched by ultra-centrifugation at 134,000 x g at 4°C for 3 hours. Virus stocks were aliquoted and stored at -80°C.

Retroviral transduction of activated human primary B cells

2 days post activation, peripheral B cells were spin-transduced with GaLVenv retrovirus. The B cell culture supernatant was removed, and diluted virus was added to the cells by gently pipetting up and down, incubated at 37˚C for 10 minutes and cells spun down in a pre-warmed centrifuge at 33˚C 800 x g for 90 minutes. After spin-transduction, the medium was changed.

Infection with EBV wildtype strain B95-8

2 days after activation, human peripheral B cells were collected, washed once with PBS, and mixed with 750 µl of pre-warmed supernatant containing EBV wildtype strain B95-8 virus particles (36,37) in a total volume of 1.5 ml. After 3 hours of incubation and rotation at 37˚C, cells were washed and seeded at 0.5 million cells/ml.

FACS staining and analysis

Cells were washed once in FACS buffer (0.5% BSA, 1 mM EDTA), stained with fluorophore-conjugated antibodies for 15 min at 4˚C, washed once and resuspended in FACS buffer containing a Live/Dead staining-dye (DAPI, PI, or LIVE/DEAD Fixable Near-IR Dead Cell Stain Kit, ThermoFisherSCIENTIFIC, Cat. No. L34976) and counting beads (CountBright absolute counting beads, Life Technologies, Cat. No. C36950), then analyzed on a LSR Fortessa (BD Biosciences) using FlowJoTM v10.6.2 Software (BD Biosciences).

Cell cycle analysis by DAPI-staining (DNA content)

Cells were washed in PBS and labeled with LIVE/DEAD Fixable Near-IR Dead Cell Stain Kit (ThermoFisherSCIENTIFIC, Cat. No. L34976) at 4˚C for 15 min to exclude dead cells. Stained cells were then washed and fixed with 1X Cytofix/Cytoperm buffer (BD, Cat. No. 554722) on ice for 20 min. Fixed cells were pelleted and washed with Perm/Wash buffer (BD, Cat. No. 554723) 2 times, before staining with 10 µg/ml DAPI in Perm/Wash buffer for 30min at room temperature. After 2 more times of washing, cells were resuspended and subjected to FACS analysis. The DAPI channel was acquired on a linear scale.

Cell growth analysis

For *in vitro* cell culture growth curves, the total number of cells at each time point was calculated based on the ratio of beads and living cells as measured by FACS, the total volume of the cell culture, and the split ratio (as recorded for the culture period). At the indicated time points, cells were collected and resuspended in 100 µl FACS buffer and analyzed by flow cytometry in the presence of 1 µl CountBright Absolute counting beads (Invitrogen, Cat. No. C36950) until 400 to 500 beads were

acquired (as gated by size and fluorescence intensity). The concentration of cells was determined as described by the manufacturer. From this value, the total cell number for each culture was calculated based on the total volume of the cell culture and the recorded split ratio (since day 3). Cells were split at a ratio of up to 1:2, when necessary, as judged by cell density and medium pH, and only when they reached more than 1 million cells/ml (resulting in newly split cells at a concentration of 1-2 million/ml). Cells were pipetted up and down softly to break up larger cell clumps. Between day 25 and 37 (the time window when LMP1 single-positive cells ceased to grow), cultures were not, or only rarely, split. Cell viability shown in Figure 3D was measured by trypan blue dye exclusion in an automated cell counter (TC20, BIO-RAD) with particles with a diameter below 5 µm excluded.

Protein extraction and Western Blot

Cells were lysed in self-made RIPA buffer (20mM Tris-HCl pH 7.5, 150mM NaCl, 1mM EDTA, 1% sodium deoxycholate, 0.1% SDS, 1% Nonidet P-40, protease inhibitor), homogenized by pipetting and incubated on ice for 30 min, then spun-down at 15,300 x g, 4°C for 15 min. Cell lysate supernatant was collected and mixed with loading buffer. The mixture was heated up to 95°C for 5 min before being frozen down at -80°C. Protein samples were separated by the SDS-PAGE and transferred to 0.22 µm PVDF-blotting membrane (0.33A per membrane, 90 minutes). Membrane with proteins were blocked with 5% skim-milk, followed by staining with primary antibody in 5% BSA and then HRP-conjugated secondary antibody in 5% skim-milk. All blocking, staining and washing steps in between were done in 1x TBST buffer (Tris-buffered saline pH 8 (Tris 20 mM, 140 mM NaCl, 0.1% Tween 20). After incubation of membrane with substrate (ECL Prime Western Blotting Detection Reagent, Cytiva, Cat. No. RPN2236), signals were detected and visualized. Western blots were quantified by densitometric analysis of bands using ImageJ 1.52q on 8-bit grey scale images. Values are given as ratio for each sample signal to actin loading control signal. Antibodies are listed in **Supplementary Table 1**.

DNA isolation and PCR amplification, cloning and analysis of IgH sequences

Cells were lysed and genomic DNA extracted using either DNA QuickExtract Solution (Lucigen, Cat. No. QE09050) or AllPrep DNA/RNA kit (QIAGEN, Cat. No. 80204) according to manufacturer's instructions. Purified genomic DNA was stored at -20°C (for less than a month) or -80°C for longer period. Using Herculase II polymerase (Herculase II Fusion Enzyme, Agilent Technologies, Cat. No. 600679), IgH variable region sequences spanning FR1, CDR1, FR2, CDR2, FR3 and CDR3 were PCR amplified using a single degenerate consensus forward primer FR1c and a mixture of JH family-specific reverse primers (55) (Aubin et al., 1995) (**Supplementary Table 2**). The PCR reaction contained 300ng heat-denatured gDNA, 0.2uM of forward primer and reverse primer-mix each, 200uM dNTP, 2% DMSO. The sequences of primer sets are shown in **Supplementary Table 2**. The PCR mix was denatured at 95°C for 3min, followed by 30 cycles of 15s denaturing at 95°C, 20s annealing at 59°C, 20s (3 min extra for the last cycle) extension at 72°C. The PCR products of around 400 bp in size were cut out of 1% agarose gels and purified (Gel and PCR Clean-up, Macherey-Nagel, Cat. No. 740609), and cloned into pJET1.2/Blunt (CloneJET PCR Cloning Kit, ThermoSCIENTIFIC, Cat. No. K1231). 1 µl of the reaction mixture was transformed into E. coli Top10, single bacterial colonies picked the next day, plasmid DNA extracted and inserts sequenced on one strand using the pJET1.2 forward primer. Sequences were analyzed with IgBlast (https://www.ncbi.nlm.nih.gov/igblast/). Low quality and non-productive VDJ sequences were excluded from further analysis, as well as the beginning of the IgH variable region covered by the degenerate FR1c forward primer.

cDNA synthesis and EBER1/2 detection

Total RNA was isolated using the miRNeasy Micro Kit (QIAGEN, Cat. No. 217084) following the manufacturer's instructions and stored at -80°C. cDNA was generated using hexamer primers according to the manufacturer's instruction (SuperScript IV First-Strand cDNA Synthesis Reaction, LifeTechnology, Cat. No. 18091050). After cDNA synthesis, the remaining RNA was degraded by RNaseH, and the cDNA stored at -80°C. Epstein-Barr virus-encoded small RNA 1 and 2 (EBER1 and EBER2) was amplified by PCR (Herculase II polymerase, 40 cycles), using synthesized cDNA as template. Primers (Jochum et al., 2012) are shown in **Supplementary Table 3**.

Growth of LMP1 and EBNA2 knock-out cells in CRISPR/Cas9-edited cell populations

SynL1E2 cell lines, frozen 60 days after transduction, were cultured for twenty days before electroporation with ribonucleoprotein (RNP) complexes as described (Seki and Rutz, 2018). crRNAs were designed by CrispRGold 1.1 (Chu et al., 2016) and purchased from IDT (**Supplementary Table 3**). To generate gRNA complexes, crRNA and tracrRNAs were combined at a 1:1 molar ratio, annealed at 95°C for 5 min and cooled down to room temperature. Cas9 protein (Alt-R S. p. Cas9 Nuclease V3) was produced in-house. RNPs were assembled at a Cas9:gRNA molar ratio of 1:2 at 25°C for 10 min before electroporation. $5x10^5$ synL1E2 cells were resuspended in 20 µl P3 electroporation buffer (Lonza, Cat. No. V4XP-3032), mixed with 50 pmol RNPs (based on Cas9 protein amounts) and electroporated using a Lonza 4D Nucleofector (program CA137, "Ramos"). After electroporation, cells were plated immediately in pre-warmed cell culture medium at 2 million cells per ml and cultured for up to twelve days. Genomic DNA was extracted from the edited cell populations on day 3 and day 10 (LMP1 targeting) or day 12 (EBNA2 targeting), as well as from non-treated, non-targeted control cells (day 10). The abundance of CRISPR/Cas9-induced frameshift mutations (knock-outs) in the edited cell populations was determined by deep sequencing of PCR amplicons using the Amplicon-EZ platform (Genewiz Germany GmbH, Azenta Life Sciences): The DNA sequence surrounding the gRNA targeting-site was amplified by PCR with PrimeSTAR GXL polymerase (TaKaRa, R050A) (401bp for LMP1 and 327bp for EBNA2; primers are shown in **Supplementary Table 3**). PCR products were purified from gels (Gel and PCR Clean-up kit, Macherey-Nagel, 740609.250), DNA quantified with Nanodrop and shipped for next generation sequencing on the Illumina platform to Genewiz Germany GmbH (Azenta Life Sciences). Merged paired sequence reads were aligned to LMP1 and EBNA2 of EBV wildtype strain B95-8. Variant detection was performed using the Genewiz proprietary Amplicon-EZ program NGS Genotyper v1.4.0 (Azenta Life Sciences).

Immunofluorescence (IF) staining of cultured B cells

1 million cells were washed with PBS, resuspended in 100 µl PBS, spun down onto glass-slides using a CytoSpin centrifuge, fixed and permeabilized for 5 minutes using a 1+1 mixture of methanol and acetone (pre-cooled on dry-ice), and air-dried overnight. For IF staining, cells were re-hydrated in PBS for 15 minutes at room temperature, followed by blocking (2% BSA in PBS for 30 minutes), incubation with primary antibodies (overnight at 4°C), secondary antibodies (2 hours), 1 µg/ml DAPI in PBS (2 minutes) and mounting (Fluoromount, Sigma, Cat. No. F4680). All steps were carried out at room temperature in 0.2% BSA/PBS, unless indicated otherwise. Slides were kept in the dark until analysis. Antibodies are listed in **Supplementary Table 2**.

Enzyme-linked Immunosorbent Assay (ELISA) for detection of secreted human Ig

96-well half-area microplates (medium-binding, Greiner Bio-One GmbH, Cat. No. 675001) were coated with capture antibody (2 µg/ml in PBS) at 4°C overnight, washed 3 times with 0.05% Tween 20 in PBS, blocked with 1% BSA in PBS for 1 hour at room temperature and washed once before over-night incubation at 4°C with standards of known concentrations or test samples serially diluted in 1% BSA/PBS. After washing, bound antibodies were detected with alkaline phosphatase-conjugated detection antibody (2 µg/ml in PBS with 1% BSA) for 2 hours at room temperature, washed and incubated with substrate. The reaction was stopped by an equal volume of 1N NaOH, when standards were well developed. Absorption at 405nm wavelength was measured using a spectrophotometer (xMark, Bio-Rad), and the sample concentration was calculated on the basis of the standard curves. Antibodies are listed in **Supplementary Table 2**.

Transplantation of cell lines into NOG mice

6-week-old female severely immunodeficient NOG mice (NOD.Cg-$Prkdc^{scid}$ $Il2rg^{tm1Sug}$/JicTac) were purchased from Taconic Biosciences (Taconic, Denmark) and handled by Experimental Pharmacological and Oncology (EPO) GmbH, Berlin Buch. Mice were acclimated for at least one week after arrival. All mouse experiments were approved by the relevant regulatory agency (Landesamt für Gesundheit und Soziales Berlin, approval number: Reg 0010/19) and conducted in compliance with the German Animal Welfare Act. Animals were kept in a 12 h light/dark cycle, at a housing temperature of 23°C. Food and water were available *ad libitum*. Selected B cell lines that had been frozen 60 days after transduction or infection were thawed, and cultured for 2 weeks before transplantation. On the day of transplantation, 7-10 million cells from each cell line were collected, pelleted and washed in PBS and spun down at 160 x g for 10 min. Cell pellets were resuspended in PBS at 100 million/ml and mixed with an equal volume of MatriGel (Corning, Cat. No. 356234). 100 µl of this cell mixture (containing 5 million cells) was injected subcutaneously into female, 2-month-old NOG mice. Three time per week, the mice were weighed and the size of the nodules at the injection sites measured with a caliper. The tumor volume was calculated using the formula 0.5 a × b2, where a and b are the long and short diameters of the tumor, respectively. After reaching a tumor volume of ≥ 1.5 cm3 animals had to be sacrificed (humane endpoint).

Tumor disassociation for FACS analysis

Subcutaneous tumors were dissected, and approximately 1/3 volume of each tumor was dissociated into single cells (GentleMACS with Tumor Disassociation Kit, Miltenyi, Cat. No. 130-095-929) following the manufacturer's instructions. For BJAB and synL1E2 tumors, GentleMACS programs "37C_h_TDK_1" and "37C_h_TDK_2" were used, respectively.

Immunofluorescence (IF) staining of cryo-sectioned tissue

1/3 of each tumor was immersed in OCT embedding matrix (Sakura, Cat. No. 4583), snap-frozen in liquid nitrogen and stored at -80°C. 7 µm thick Cryo-sections were cut (ThermoFisherSCIENTIFIC, CryoStar NX70) and placed on polysine-coated glass slides, and either stained directly or stored at -80°C. Cryo-sections were fixed using 4% para-formaldehyde at room temperature for 30 minutes and in case of intra-nuclear staining additionally permeabilized with 0.5% Triton/PBS for 25 minutes at room temperature. Following 3 times washing steps with PBS at room temperature, tissue sections were blocked with 2% BSA in PBS for 1 hour and incubated with primary antibodies (in 0.2% BSA in PBS) for 1 hour at 37°C. If the primary antibodies were not fluorophore-conjugated, sections

stained with primary antibody were then incubated with secondary antibodies for 2 hours at 37°C. After 3 times washing with PBS, sections were incubated with DAPI (2 μg/ml in PBS) for 10 minutes, sealed and kept in the dark until analysis. Antibodies are listed in **Supplementary Table 2**.

Quantification of genomic mouse and human DNA in tumor samples

Mouse and human genomic DNA for quantification standards was isolated from splenocytes of C57BL/6 wildtype mice and the synL1E2-13 cell line, respectively. Mouse and human gDNA samples were mixed at defined molar ratios (10:0, 8:2, 5:5, 2:8, 0:10). Tumor gDNA was isolated from cryo-sections of 60 μm thick, using QIAamp DNA Micro Kit (QIAGEN, Cat. No. 56304). The single copy gene Hira was amplified by PCR (Herculase II polymerase, 30 cycles). Primers are shown in **Supplementary Table 3**. The PCR products were separated (mouse: 590bp; human: 574bp) on a 2.5% agarose gel, and quantified using ImageJ.

Cytokine profiling

Cell culture supernatant from synL1E2 cell lines, conventional LCLs and BJAB lines was collected after 3 days (72 hours) of *in vitro* culture after seeding 1 million cells /ml, centrifuged at 800 x g to remove cell debris, frozen and stored at -80°C until analysis. Eight cytokines (IL1β, IFN-γ, TNF-α, IL12p70, IL-17A, IL-18, IL-23, IL-33) and five chemokines (CXCL8 (IL-8), CXCL10 (IP-10), CCL5 (RANTES), CXCL1 (GROα), CCL (MIP-1β)), respectively, were quantified according to the manufacturer's instructions in an 8-plex and 5-plex format, using two LEGENDplex Multi-Analyte Flow Assay Kits with the corresponding Mix and Match subpanels from Human Inflammation Panel 1 (BioLegend, Cat. No. 740809) and Human Proinflammatory Chemokine Panel (BioLegend, Cat. No. 740985). Briefly, 25 μl of a four-fold dilution series of standards and undiluted supernatants were incubated with pre-mixed 8-plex or 5-plex capture beads for 2 hours at room temperature on a shaker. Then detection antibody-mix was added and incubated for 1 hour at room temperature. After 30 min incubation with substrate and washing, the fluorescence intensity of the beads was determined by flow cytometry on a LSR Fortessa (BD Biosciences). Cytokine and chemokine concentrations were calculated based on the standard curves, using the cloud-based LEGENDplex Data Analysis Software Suite (version 2023-02-15). For the three chemokines CCL4 (MIP1β), CCL5 (RANTES) and CXCL10 (IP-10), several measurements exceeded the highest standard concentrations. These values were set to the highest standard concentration plus 1 pg/ml.

Supplementary Tables

Supplementary Table 1. Nucleotide sequence of EBNA2 CDS after optimization for expression on the transcriptional and translational level.

ATGCCCACCTTCTATCTGGCTCTGCACGGCGGACAGACCTACCACCTGATCGTGGATACAGACAGCCTGG

GCAACCCTAGCCTGAGCGTGATCCCTAGCAACCCCTACCAAGAGCAGCTGAGCGACACACCTCTGATCC

CTCTGACAATCTTCGTGGGCGAGAACACAGGCGTTCCACCTCCATTGCCTCCACCGCCACCGCCGCCTCC

ACCACCTCCGCCTCCACCTCCGCCACCACCGCCACCGCCTCCGCCGCCACCACCTTCTCCACCGCCGCCA

CCTCCACCACCACCACCGCCTCAAAGAAGAGATGCCTGGACACAAGAGCCCAGTCCTCTGGATAGAGAT

CCCCTGGGATACGATGTCGGACACGGACCTCTGGCCTCTGCCATGAGAATGCTGTGGATGGCCAACTAC

ATCGTGCGGCAGTCTAGAGGCGACAGAGGCCTGATTCTGCCTCAGGGACCTCAGACAGCTCCACAGGCT

AGACTGGTGCAGCCTCATGTGCCTCCACTCAGACCAACCGCTCCTACCATCCTGTCTCCACTGAGCCAGC

CTAGACTGACACCTCCTCAGCCTCTGATGATGCCTCCTAGGCCTACACCTCCTACACCACTGCCTCCAGC

TACACTGACAGTGCCTCCAAGACCAACCAGGCCTACCACACTTCCTCCAACACCTCTGCTGACTGTGCTG

CAGAGGCCTACAGAACTGCAGCCCACACCATCTCCACCTAGAATGCATCTGCCAGTGCTGCACGTGCCC

GACCAGTCTATGCATCCTCTGACACACCAGAGCACCCCTAACGACCCTGACAGCCCTGAGCCTAGAAGC

CCCACCGTGTTCTACAACATCCCTCCTATGCCACTGCCACCTAGTCAGTTGCCACCTCCTGCTGCTCCAGC

TCAACCTCCTCCTGGCGTGATCAATGACCAGCAGCTGCACCATCTGCCATCTGGACCTCCATGGTGGCCT

CCAATCTGTGACCCTCCACAGCCTTCTAAGACCCAGGGACAGAGCAGAGGACAGTCCCGTGGTAGAGGC

CGTGGACGTGGAAGAGGTAGAGGCAAGGGCAAGAGCAGAGACAAGCAGAGAAAACCTGGCGGCCCTTG

GAGGCCTGAGCCAAACACATCTAGCCCTAGCATGCCTGAGCTGAGCCCTGTGCTGGGACTGCATCAAGG

ACAAGGTGCCGGCGATTCTCCTACTCCTGGACCTTCTAACGCCGCTCCTGTGTGCAGAAACTCTCACACC

GCCACACCTAACGTGTCCCCAATCCACGAGCCTGAGAGCCACAATAGCCCCGAGGCTCCTATCCTGTTTC

CTGACGACTGGTATCCTCCTAGCATCGACCCTGCCGACCTGGACGAGAGCTGGGATTACATCTTCGAGAC

AACCGAGTCTCCCAGCAGCGACGAGGATTACGTGGAAGGACCTAGCAAGAGGCCCAGACCTAGCATCC

AA

Supplementary Table 2. List of reagents. FC, flow cytometry; IF, Immunofluorescence; WB, Western blot

Antibodies	Supplier, Cat. No., RRID	Clone	Dilution or concentration
FITC anti-human CD19	BioLegend, 302206, AB_314236	HIB19	FC, 1:200
APC anti-human CD19	BioLegend, 302212, AB_314242	HIB19	FC, 1:200
Brilliant Violet 605™ anti-human CD19	BioLegend, 302243, AB_2562014	HIB19	FC, 1:200
Brilliant Violet 785™ anti-human CD19	BioLegend, 302240, AB_2563442	HIB19	FC, 1:200
Brilliant Violet 650™ anti-human CD20	BioLegend, 302336, AB_2563806	2H7	FC, 1:200
BV650 Mouse Anti-Human CD138	BDBioscience, 743500, AB_2741548	MI15	FC, 1:200
PE/Cy7 anti-human CD21	BioLegend, 354912, AB_2561577	Bu32	FC, 1:200
APC anti-human CD23	BioLegend, 338514, AB_1501112	EBVCS-5	FC, 1:200
BV650 Mouse Anti-Human CD27	BDBioscience, 564894, AB_2739004	M-T271	FC, 1:200
CD27-PE, human	Miltenyi, 130-114-156, AB_2751157	M-T271	FC, 1:200
PerCP/Cyanine5.5 anti-human CD54	BioLegend, 353120, AB_2715948	HA58	FC, 1:200
Brilliant Violet 785™ anti-human CD95 (Fas)	BioLegend, 305646, AB_2629742	DX2	FC, 1:200
PerCP/Cyanine5.5 anti-human HLA-DR, DP, DQ	BioLegend, 361710, AB_2750312	Tü39	FC, 1:200
BV786 Mouse Anti-Human CD38	BDBioscience, 741040, AB_2740656	HB7	FC, 1:200

IgD Antibody, anti-human, PE-Vio® 770	Miltenyi, 130-098-584, AB_2659776	IgD26	FC, 1:200
PerCP/Cyanine5.5 anti-human IgM	BioLegend, 314512, AB_2076098	MHM-88	FC, 1:200
IgA Antibody, anti-human, APC-Vio® 770	Miltenyi, 130-113-999, AB_2733153	IS11-8E10	FC, 1:200
IgA Antibody, anti-human, PE-Vio770	Miltenyi, 130-113-477, AB_2751114	IS11-8E10	FC, 1:200
PE/Cy7 anti-human Ig light chain λ	BioLegend, 316623, AB_2687263	MHL-38	FC, 1:200
APC anti-human Ig light chain κ	BioLegend, 316510, AB_493615	MHK-49	FC, 1:200
APC anti-human HLA-A, B, C	BioLegend, 311410, AB_314879	W6/32	FC, 1:200
Brilliant Violet 605™ anti-mouse CD45	Biolegend, 103140, AB_2562342	30-F11	FC, 1:200
PE/Cyanine7 anti-mouse Ly-6G	BioLegend, 127618, AB_1877261	1A8	FC, 1:200
FITC anti-mouse Ly-6C	BioLegend, 128006, AB_1186135	HK1.4	FC, 1:200
Brilliant Violet 421™ anti-mouse NK-1.1	BioLegend, 108731, AB_10895916	PK136	FC, 1:200
Allophycocyanin (APC) AffiniPure F(ab')$_2$ Fragment Goat Anti-Human IgG, Fcγ fragment specific	Dianova Jackson, 109-136-170, AB_2337695	Polyclonal	FC, 1:800
Alexa Fluor®488 anti-human Ki67	BioLegend, 350532, AB_2564169	Ki-67	IF, 5 µg/ml
Alexa Fluor®647 anti-mouse CD45	BioLegend, 103124, AB_493533	30-F11	IF, 10 µg/ml
Alexa Fluor®647 anti-mouse/human CD11b	BioLegend, 101218, AB_389327	M1/70	IF, 10 µg/ml
Alexa Fluor®647 anti-mouse Ly-6C	BioLegend, 128010, AB_1236550	HK1.4	IF, 10 µg/ml

Alexa Fluor®594 anti-mouse Ly-6G	BioLegend, 127636, AB_2563207	1A8	IF, 10 µg/ml
Anti-EBNA2	abcam, ab90543	PE2	WB, 5 ng/µl;<br>IF, 10 ng/µl
Anti-LMP1, Mouse, hybridoma supernatant	(Mann et al., 1985)	S12	WB, undiluted
Anti-LMP2A	AbsoluteAntibody, Ab01294-7.1	4B7	WB, 1:500<br>IF, 1:50
Unconjugated Goat anti-human IgG<br>Capture antibody for ELISA	Southern Biotech, 2040-01, AB_2795640	Polyclonal	2 µg/ml
Unconjugated Goat anti-human IgM,<br>Capture antibody for ELISA	Southern Biotech, 2020-01, AB_2795599	Polyclonal	2 µg/ml
Unconjugated Goat anti-human IgA,<br>Capture antibody for ELISA	Southern Biotech, 2050-01, AB_2795701	Polyclonal	2 µg/ml
Unconjugated human IgG,<br>Standard for ELISA	Southern Biotech, 0150-01, AB_2794075	Polyclonal	
Isotype control human IgM,<br>Standard for ELISA	Life Technologies, 31146, AB_844400	Polyclonal	
Unconjugated human IgA,<br>Standard for ELISA	BIOZOL Diagnostica, SBA-0155K-01, AB_2794089	Polyclonal	
AP-coupled Goat anti-human IgG,<br>Detection antibody for ELISA	Southern Biotech, 2040-04, AB_2795643	Polyclonal	2 µg/ml
AP-coupled Goat anti-human IgM,<br>Detection antibody for ELISA	Southern Biotech, 2020-04, AB_2795602	Polyclonal	2 µg/ml
AP-coupled Goat anti-human IgA,<br>Detection antibody for ELISA	Southern Biotech, 2050-05, AB_2687526	Polyclonal	2 µg/ml

Supplementary Table 3. List of oligonucleotide primers.

**Primers for VDJ-PCR**	
huIg-commonVH-F	AGGTGCAGCTGSWGSAGTCDGG
huIg-JH1_2_4_5-R	ACCTGAGGAGACGGTGACCAGGGT
huIg-JH3-R	TACCTGAAGAGACGGTGACCATTGT
huIg-JH6-R	ACCTGAGGAGACGGTGACCGTGGT
**Primers for detection of EBER1/2 and GAPDH**	
EBER1_F	GACCTACGCTGCCCTAGAGGTTTTGC
EBER1_R	CCAGCTGGTACTTGACCGAAGACG
EBER2_F	GGACAGCCGTTGCCCTAGTGG
EBER2_R	AGCGGACAAGCCGAATACCCTTC
GAPDH_F	ATGCCTCCTGCACCACCAAC
GAPDH_R	GGCCATCCACAGTCTTCTGG
**Oligonucleotides for CIRPSR knock out**	
crLMP1	CGCCGGCTCCACTCACGAGC
crEBNA2	TACATCGTGCGGCAGTCTAG
**Primers for LMP1 and EBNA2 targeted site genomic amplicon sequencing**	
LMP1_F	ACACTCTTTCCCTACACGACGCTCTTCCGATCTTTAATCTGGATGTATTACCA
LMP1_R	GACTGGAGTTCAGACGTGTGCTCTTCCGATCTGCAGAGTCGCTAGGGCTATG
EBNA2_F	ACACTCTTTCCCTACACGACGCTCTTCCGATCTAGATGCCTGGACACAAGAGC
EBNA2_R	GACTGGAGTTCAGACGTGTGCTCTTCCGATCTGTCAGTGTAGCTGGAGGCAG
**Primers for quantification of mouse and human genomic DNA content**	
PrForGT_HIRA-1fw	GGCCGCACCTCCAAG
PrForGT_HIRA-1re	ACCTGGTTCTCTAGGTAGGCCAG

Supplementary References

Aubin, J., Davi, F., Nguyen-Salomon, F., Leboeuf, D., Debert, C., Taher, M., Valensi, F., Canioni, D., Brousse, N., Varet, B., 1995. Description of a novel FR1 IgH PCR strategy and its comparison with three other strategies for the detection of clonality in B cell malignancies. Leukemia 9, 471–9.

Chu, V.T., Graf, R., Wirtz, T., Weber, T., Favret, J., Li, X., Petsch, K., Tran, N.T., Sieweke, M.H., Berek, C., Kühn, R., Rajewsky, K., 2016. Efficient CRISPR-mediated mutagenesis in primary immune cells using CrispRGold and a C57BL/6 Cas9 transgenic mouse line. Proc National Acad Sci 113, 12514–12519. https://doi.org/10.1073/pnas.1613884113

Jochum, S., Ruiss, R., Moosmann, A., Hammerschmidt, W., Zeidler, R., 2012. RNAs in Epstein–Barr virions control early steps of infection. Proc. Natl. Acad. Sci. 109, E1396–E1404. https://doi.org/10.1073/pnas.1115906109

Mann, K.P., Staunton, D., Thorley-Lawson, D.A., 1985. Epstein-Barr virus-encoded protein found in plasma membranes of transformed cells. J. Virol. 55, 710–720. https://doi.org/10.1128/jvi.55.3.710-720.1985

Seki, A., Rutz, S., 2018. Optimized RNP transfection for highly efficient CRISPR/Cas9-mediated gene knockout in primary T cells. J. Exp. Med. 215, 985–997. https://doi.org/10.1084/jem.20171626

Supplementary Figures

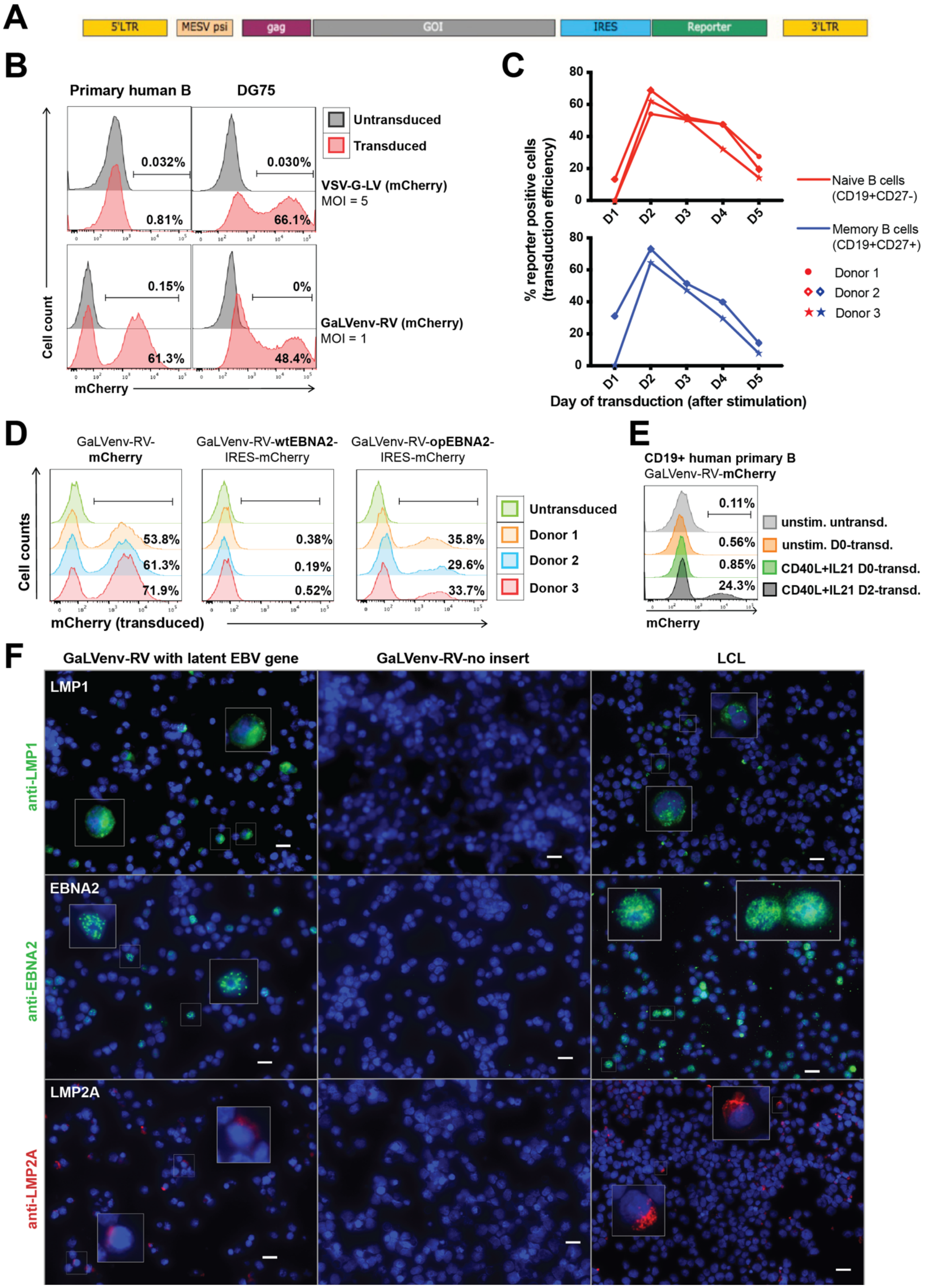

Supplementary Figure 1 (Supporting data for **Figure 1**) GaLVenv-Retroviral transduction enables expression of EBV latent proteins LMP1, LMP2A and EBNA2 in human primary B cells. **(A)** Bicistronic provirus integrated into the host genome after transduction with GaLVenv-RV. The 5' LTR serves as promoter and the gene-of-interest (GOI) is followed by an internal ribosome entry site (IRES) and a fluorescent reporter (GFP or mCherry). **(B)** Primary human B cells can be efficiently transduced with GaLVenv-RV. Human peripheral B cells (left) or the human Burkitt lymphoma line DG75 (right) were transduced with lentivirus pseudotyped with VSV-G (VSV-G-LV, upper row) or retrovirus pseudotyped with GaLVenv (GaLVenv-RV, lower row) and analyzed four days after transduction (no inserts). The fraction of mCherry reporter positive cells was determined by flow cytometry. **(C)** GaLVenv-RV transduction efficiency in human peripheral B cells peaks two days after B cell isolation and stimulation (no inserts). Naïve (CD27-) and memory (CD27+) B cell subpopulations were isolated and transduced at an MOI of 1 at the indicated time points. The fraction of reporter positive cells was determined by flow cytometry four days after transduction. **(D)** An expression-optimized version of EBNA2 shows improved overall expression in primary human B cells. Human B cells were transduced with GaLVenv-RV carrying no insert, wildtype EBNA2 (wtEBNA2) or optimized EBNA2 (opEBNA2). Untransduced B cells from one donor are shown as a representative negative control. The fraction of mCherry reporter-positive cells was determined by flow cytometry. Gating is indicated. **(E)** Transduction of primary B cells with GaLVenv-RV requires activation with CD40L and IL-21. Unstimulated B cells (orange) were refractory to transduction, in contrast to B cells 2 days after stimulation (dark grey). Cells were analyzed by flow cytometry 4 days after B cell isolation (light grey) or GaLVenv-RV transduction (orange, green, dark grey). **(F)** Immunofluorescence staining four days after transduction reveals expected intracellular protein locations for LMP1 (perinuclear), EBNA2 (nuclear) and LMP2A (perinuclear). B cells were fixed with acetone/methanol and stained with antibodies specific for LMP1, LMP2A or EBNA2, respectively, as indicated on the left. Cells transduced with GFP or mCherry only were used as negative controls (middle column) and LCLs as positive controls (right column). Images are representative for three different donors. RV, retroviruses. Size bar, 20 µm.

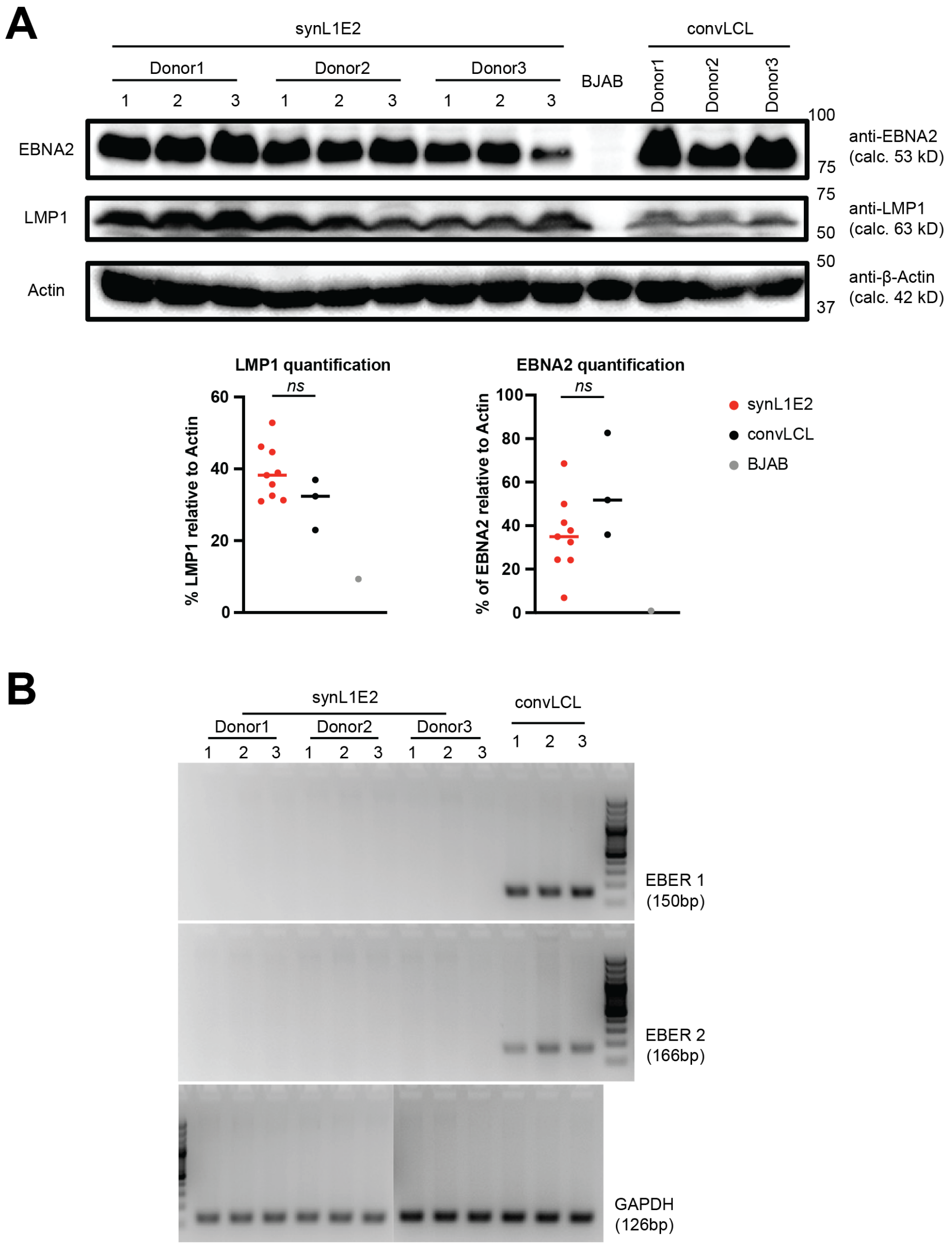

Supplementary Figure 2 (Supporting data for **Figure 2**) SynL1E2 cell lines express LMP1 and EBNA2 proteins in the absence of EBV infection. **(A)** Western blots 60 days after LMP1 and EBNA2 double transduction. Three outgrown synL1E2 cell lines derived from three EBV-negative donors were lysed and proteins were separated by SDS-PAGE. Wildtype BJAB cells served as negative, and three convLCLs as positive controls. calc., calculated. Lower panel: Quantification by densitometric analysis of bands relative to actin loading controls. Each dot represents one sample, bars indicate median. Unpaired, two-tailed t-test with Welch's correction. LMP1, $p = 0.17$, EBNA2, $p = 0.26$. **(B)** SynL1E2 cell lines are free from EBV-infection. Reverse transcriptase PCR was performed 60 days after LMP1/EBNA2-transduction to detect non-coding RNAs EBER1 and EBER2, using whole RNA isolated from cell lysate as template. GAPDH was amplified to serve as loading control. EBER transcription was not detected in synL1E2 lines.

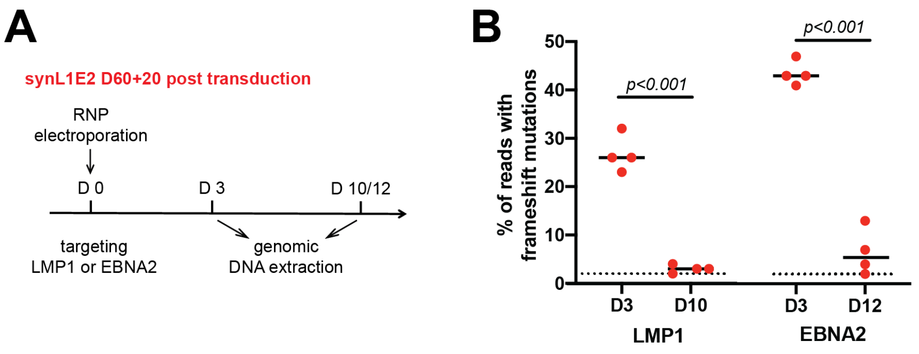

Supplementary Figure 3 (Supporting data **for Figure 2**). Continuous growth of synL1E2 cell lines is dependent on LMP1 and EBNA2 expression. **(A)** Experimental scheme. In order to disrupt LMP1 or EBNA2 genes, synL1E2 cell lines were electroporated with Cas9:gRNA RNP complexes at day 80 post-transduction (in discontinuous culture: 60 days culture plus 20 days re-culture after freezing and thawing). Genomic DNA was isolated from the edited cell populations at the indicated time points. The frequency of alleles carrying frameshift (knock-out) mutations was determined by amplicon deep sequencing of the targeted gene regions. **(B)** Percentage of reads with indels leading to frameshift (knock-out) mutations for four independent synL1E2 cell lines (n=4). Each dot represents one independent cell line, bars indicate median. Paired, two-tailed t-test, *p* values are indicated. Dashed lines indicate the background in non-treated control cells on day 10 (n=1).

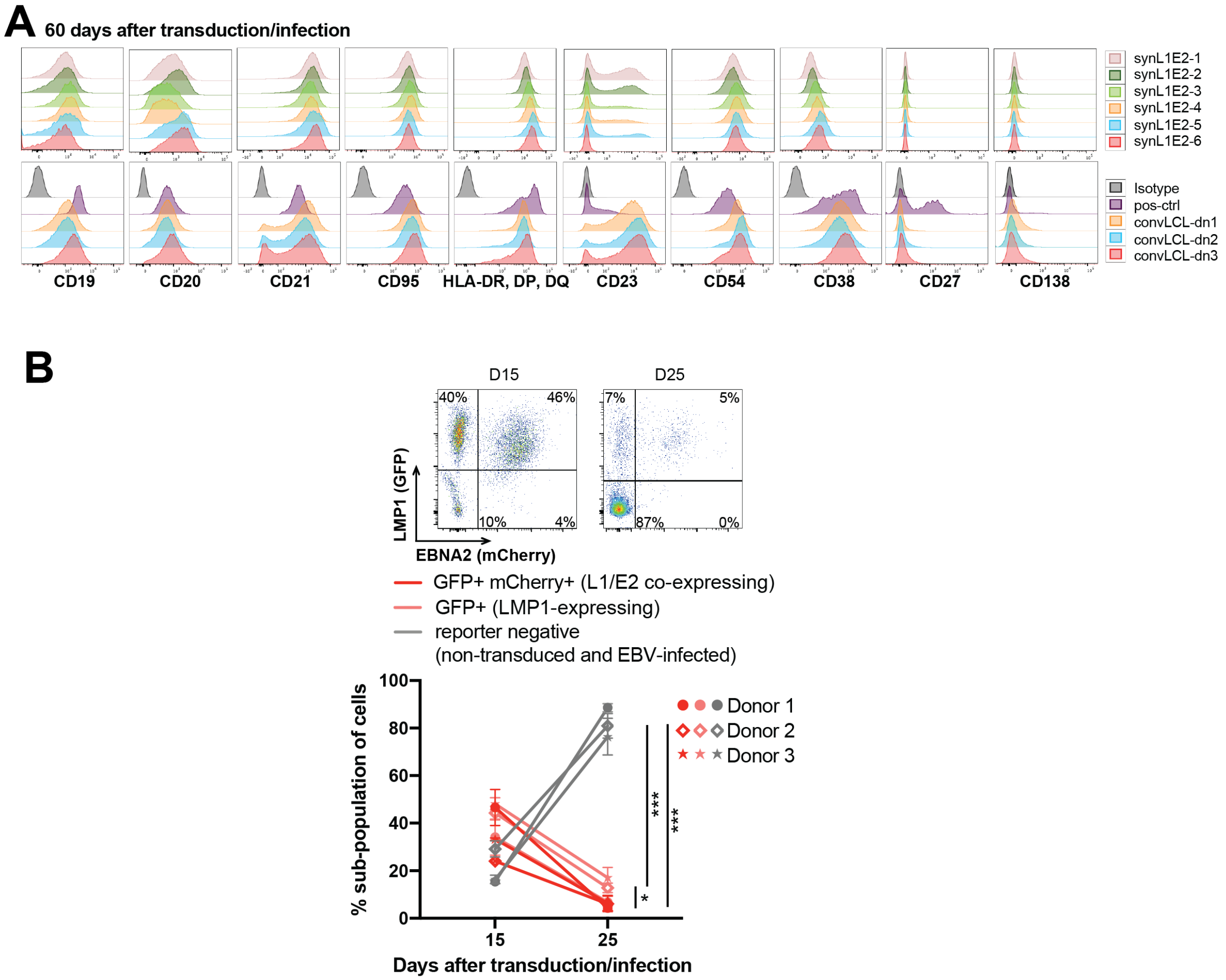

A
60 days after transduction/infection
synL1E2-1
synL1E2-2
synL1E2-3
synL1E2-4
synL1E2-5
synL1E2-6
Isotype
pos-ctrl
convLCL-dn1
convLCL-dn2
convLCL-dn3
CD19
CD20
CD21
CD95
HLA-DR, DP, DQ
CD23
CD54
CD38
CD27
CD138
B
D15
D25
40%
46%
10%
4%
7%
5%
87%
0%
LMP1 (GFP)
EBNA2 (mCherry)
GFP+ mCherry+ (L1/E2 co-expressing)
GFP+ (LMP1-expressing)
reporter negative
(non-transduced and EBV-infected)
% sub-population of cells
Donor 1
Donor 2
Donor 3
***
***
*
Days after transduction/infection

Supplementary Figure 4 (Supporting data for **Figure 3**) Synthetic LMP1/EBNA2 expressing B cells are phenotypically similar to conventional LCLs, but have a more restricted proliferative capacity. **(A)** Surface marker profiles of human B cells 60 days after transduction with LMP1 and EBNA2 (synL1E2) or infection with EBV wildtype strain B95-8 (convLCL). Antibodies with matching isoptypes were used as negative controls. As positive controls (pos-ctrl) we used peripheral B cells either on the day of isolation (unstimulated; CD21, CD23, CD19, CD20), or peripheral B cells stimulated by CD40L/IL-21 double positive feeder cells for 5 days (HLA-II) or 10 days (CD27, CD38, CD54, CD95, CD138). The cell line CAA is shown as a reference; it was generated by infecting unstimulated CD27+/IgD- B cells (freshly isolated from blood of a healthy donor) with EBV strain B95-8. **(B)** Outgrowth of EBV-infected over in L1/E2-expressing primary B cells in competitive co-culture. 15 days after infection or transduction, non-transduced B cells infected with EBV wildtype strain B95-8 were put in co-culture with double-transduced, LMP1/EBNA2-coexpressing B cells (see Fig. 2A for experimental set up). Growth was monitored by analyzing cellular subpopulations by flow cytometry. Upper panels: Flow cytometry analysis of B cells identifies three subpopulations: double-transduced (mCherry and GFP double-positive), single transduced (GFP-positive) and non-transduced (reporter-negative, grey). Lower Panel: Change of cell subpopulations over time for duplicate cultures of cells from three donors. EBV-infected, non-transduced B cells overtook the culture by day 25. Data points represent mean with range (n=2). Paired repeated measurements one-way ANOVA (with Geisser-Greenhouse correction) and Tukey multiple comparison test (day 25), $p \leq 0.001$ (***), $p = 0.02$ (*).

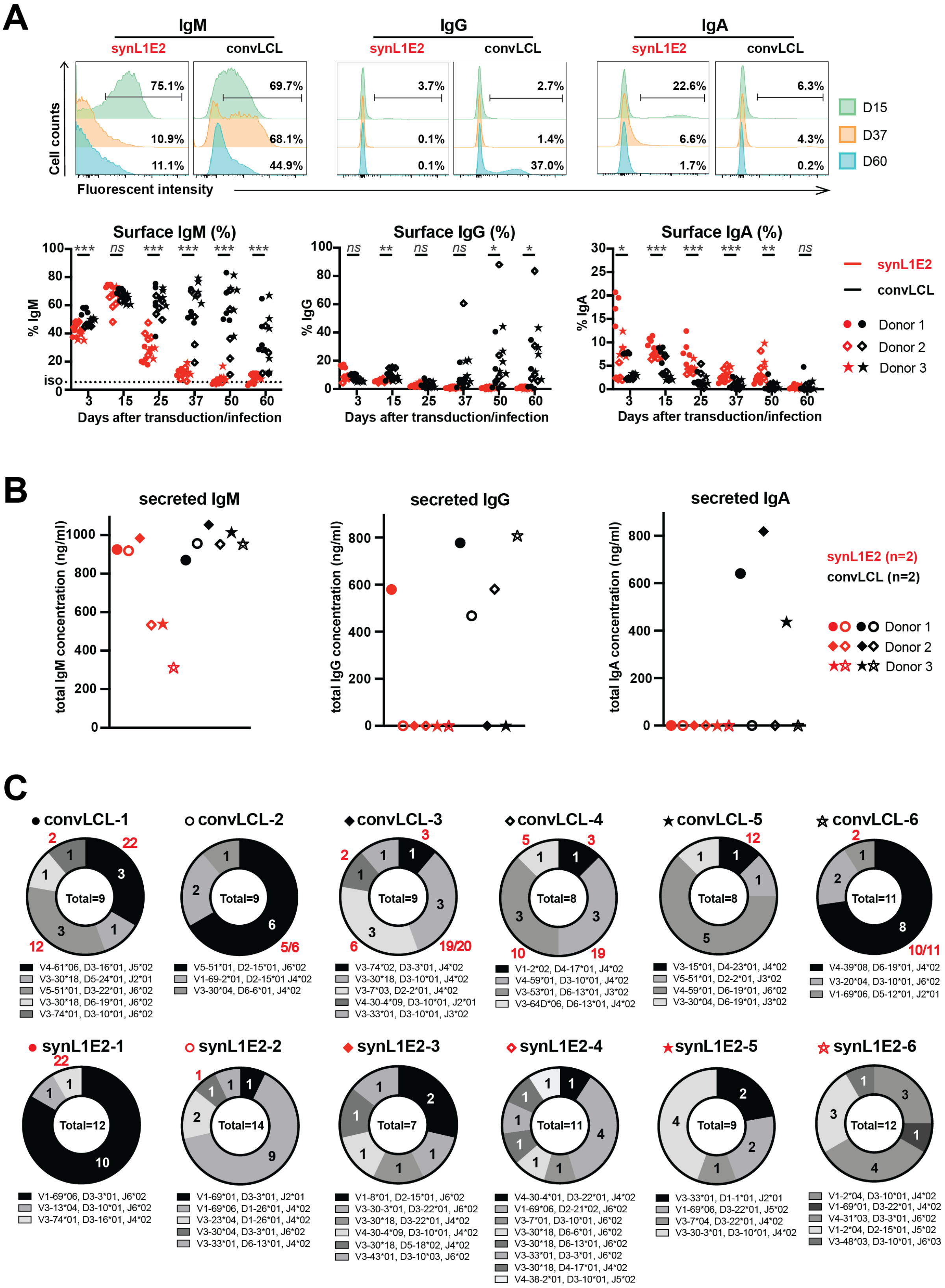
A
IgM
synL1E2
convLCL
IgG
synL1E2
convLCL
IgA
synL1E2
convLCL
75.1%
10.9%
11.1%
69.7%
68.1%
44.9%
3.7%
0.1%
0.1%
2.7%
1.4%
37.0%
22.6%
6.6%
1.7%
6.3%
4.3%
0.2%
D15
D37
D60
Cell counts
Fluorescent intensity
Surface IgM (%)
Surface IgG (%)
Surface IgA (%)
% IgM
% IgG
% IgA
iso
Days after transduction/infection
synL1E2
convLCL
Donor 1
Donor 2
Donor 3
B
secreted IgM
secreted IgG
secreted IgA
total IgM concentration (ng/ml)
total IgG concentration (ng/ml)
total IgA concentration (ng/ml)
synL1E2 (n=2)
convLCL (n=2)
Donor 1
Donor 2
Donor 3
C
convLCL-1
Total=9
V4-61*06, D3-16*01, J5*02
V3-30*18, D5-24*01, J2*01
V5-51*01, D3-22*01, J6*02
V3-30*18, D6-19*01, J6*02
V3-74*01, D3-10*01, J6*02
convLCL-2
Total=9
V5-51*01, D2-15*01, J6*02
V1-69-2*01, D2-15*01 J4*02
V3-30*04, D6-6*01, J4*02
convLCL-3
Total=9
V3-74*02, D3-3*01, J4*02
V3-30*18, D3-10*01, J4*02
V3-7*03, D2-2*01, J4*02
V4-30-4*09, D3-10*01, J2*01
V3-33*01, D3-10*01, J3*02
convLCL-4
Total=8
V1-2*02, D4-17*01, J4*02
V4-59*01, D3-10*01, J4*02
V3-53*01, D6-13*01, J3*02
V3-64D*06, D6-13*01, J4*02
convLCL-5
Total=8
V3-15*01, D4-23*01, J4*02
V5-51*01, D2-2*01, J3*02
V4-59*01, D6-19*01, J6*02
V3-30*04, D6-19*01, J3*02
convLCL-6
Total=11
V4-39*08, D6-19*01, J4*02
V3-20*04, D3-10*01, J6*02
V1-69*06, D5-12*01, J2*01
synL1E2-1
Total=12
V1-69*06, D3-3*01, J6*02
V3-13*04, D3-10*01, J6*02
V3-74*01, D3-16*01, J4*02
synL1E2-2
Total=14
V1-69*01, D3-3*01, J2*01
V1-69*06, D1-26*01, J4*02
V3-23*04, D1-26*01, J4*02
V3-30*04, D3-3*01, J6*02
V3-33*01, D6-13*01, J4*02
synL1E2-3
Total=7
V1-8*01, D2-15*01, J6*02
V3-30-3*01, D3-22*01, J6*02
V3-30*18, D3-22*01, J4*02
V4-30-4*09, D3-10*01, J4*02
V3-30*18, D5-18*02, J4*02
V3-43*01, D3-10*03, J6*02
synL1E2-4
Total=11
V4-30-4*01, D3-22*01, J4*02
V1-69*06, D2-21*02, J6*02
V3-7*01, D3-10*01, J6*02
V3-30*18, D6-6*01, J6*02
V3-30*18, D6-13*01, J6*02
V3-33*01, D3-3*01, J6*02
V3-30*18, D4-17*01, J4*02
V4-38-2*01, D3-10*01, J5*02
synL1E2-5
Total=9
V3-33*01, D1-1*01, J2*01
V1-69*06, D3-22*01, J5*02
V3-7*04, D3-22*01, J4*02
V3-30-3*01, D3-10*01, J4*02
synL1E2-6
Total=12
V1-2*04, D3-10*01, J4*02
V1-69*01, D3-22*01, J4*02
V4-31*03, D3-3*01, J6*02
V1-2*04, D2-15*01, J5*02
V3-48*03, D3-10*01, J6*03

Supplementary Figure 5 (Supporting data for **Figure 3**) Ig expression and somatic hypermutation profiles of synL1E2 cell lines. **(A)** Upper panel: Histograms depicting the median fluorescence intensity (MFI) of surface Ig for one representative cell line each, as measured by flow cytometry. Lower panel: percentage of surface IgM, IgG and IgA expression. Unpaired repeated measurements two-way ANOVA (with Geisser-Greenhouse correction) with Bonferroni multiple comparisons test, $p \leq 0.001$ (***), $p \leq 0.01$ (**), $p \leq 0.05$ (*), ns = not significant. (n=15; each dot represents one independent cell line). **(B)** Antibody secretion by synL1E2 cell lines and convLCLs as measured by ELISA (n=6; each dot corresponds to the average of duplicate cultures of independent cell lines that were cultured for 3 days). **(C)** Sequence analysis of the cell lines analyzed in Figure 4B. 50 days after transduction or infection, respectively, rearranged immunoglobulin heavy chain variable gene segments were amplified from genomic DNA and subcloned. 16 bacterial colonies were sequenced per cell line. Sequences were compared to the IMGT database using IgBLAST (Ye et al 2013 NAR). Non-productive VDJ and low-quality sequences were excluded. Pie charts depict the total number of productive rearrangements. Sequences with the same VDJ rearrangement were considered clonal and are presented in the same color, with the number of clonal sequences indicated in the pie section. In case of mismatches to the most closely related germline V gene sequence, the absolute number of mismatches is given next to the pie section (in red). "/" indicates intraclonal variation with respect to the number of mismatches.

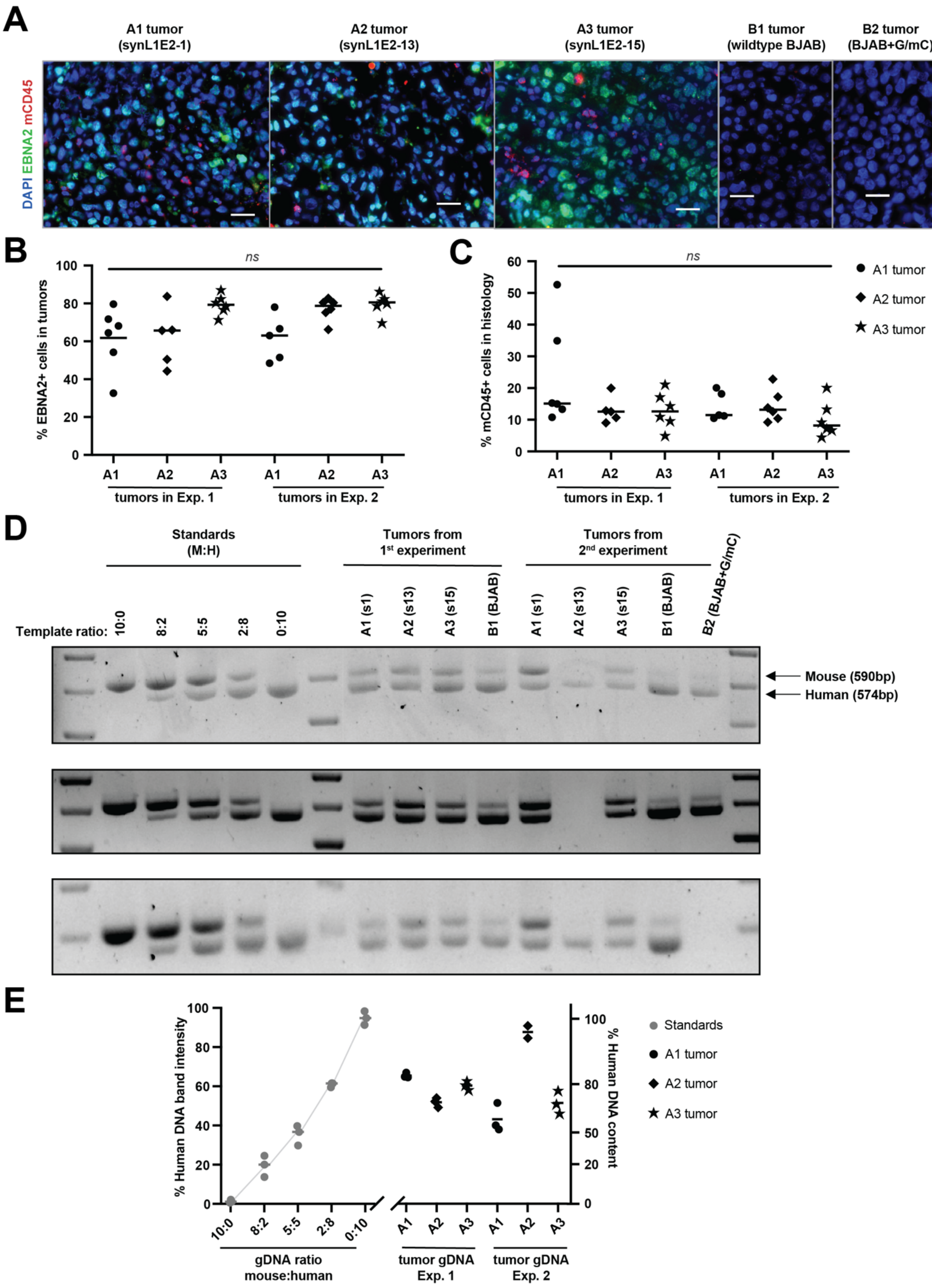
A
A1 tumor (synL1E2-1)
A2 tumor (synL1E2-13)
A3 tumor (synL1E2-15)
B1 tumor (wildtype BJAB)
B2 tumor (BJAB+G/mC)
DAPI EBNA2 mCD45
B
% EBNA2+ cells in tumors
ns
A1 A2 A3 A1 A2 A3
tumors in Exp. 1
tumors in Exp. 2
C
% mCD45+ cells in histology
ns
A1 tumor
A2 tumor
A3 tumor
tumors in Exp. 1
tumors in Exp. 2
D
Standards (M:H)
Tumors from 1st experiment
Tumors from 2nd experiment
Template ratio: 10:0 8:2 5:5 2:8 0:10
A1 (s1) A2 (s13) A3 (s15) B1 (BJAB)
A1 (s1) A2 (s13) A3 (s15) B1 (BJAB) B2 (BJAB+G/mC)
Mouse (590bp)
Human (574bp)
E
% Human DNA band intensity
% Human DNA content
Standards
A1 tumor
A2 tumor
A3 tumor
10:0 8:2 5:5 2:8 0:10
gDNA ratio mouse:human
tumor gDNA Exp. 1
tumor gDNA Exp. 2

Supplementary Figure 6 (Supporting data for **Figure 4**) LMP1/EBNA2-coexpressing synL1E2 cells grow *in vivo* in NOG mice. **(A)** Immunofluorescence of representative cryosections from tumors (Experiment 2). Seven µm thick sections were fixed, permeabilized and stained with DAPI (blue; nuclei), anti-mouse CD45 (leukocytes; red) and anti-EBNA2 (LMP1/EBNA2 expressing human B cells; green) antibodies. Size bar, 20 µm. **(B** and **C)** Quantification of EBNA2- and mouse CD45 (mDC45)-expressing cells in synL1E2 tumor cryosections. Each data point represents cell counts from one micrograph (two cryosections per tumor). Bars indicate median. Unpaired one-way ANOVA with Tukey multiple comparisons test, ns = not significant (p = 0.12). **(B)** More than 60% of all cells expressed EBNA2. **(C)** All tumors showed infiltration by mCD45-positive cells (mouse leukocytes). **(D)** PCR-based quantification of human versus mouse genomic DNA in synL1E2 tumor cryo-sections. Agarose gelelectrophoresis of PCR products. Using the same primer pair, the single copy gene *hira* was amplified from genomic DNA and the PCR products quantified. gDNA from mouse splenocytes and *in vitro* cultured synL1E2 cells mixed in pre-defined ratios were used as standards. For more details, see Materials and Methods. **(E)** PCR-based quantification of human versus mouse genomic DNA in synL1E2 tumor cryo-sections, corresponding to **Supplementary Figure 6D**. Mouse splenocytes and cultured synL1E2 cells were lysed, and the gDNA was mixed at pre-defined ratios and were used as standards (grey; left) for PCR with the same, cross-species-reactive primer pair that was used for tumor cryosections (right). Each data point corresponds to an independent PCR reaction on the same template. Bars indicate median.

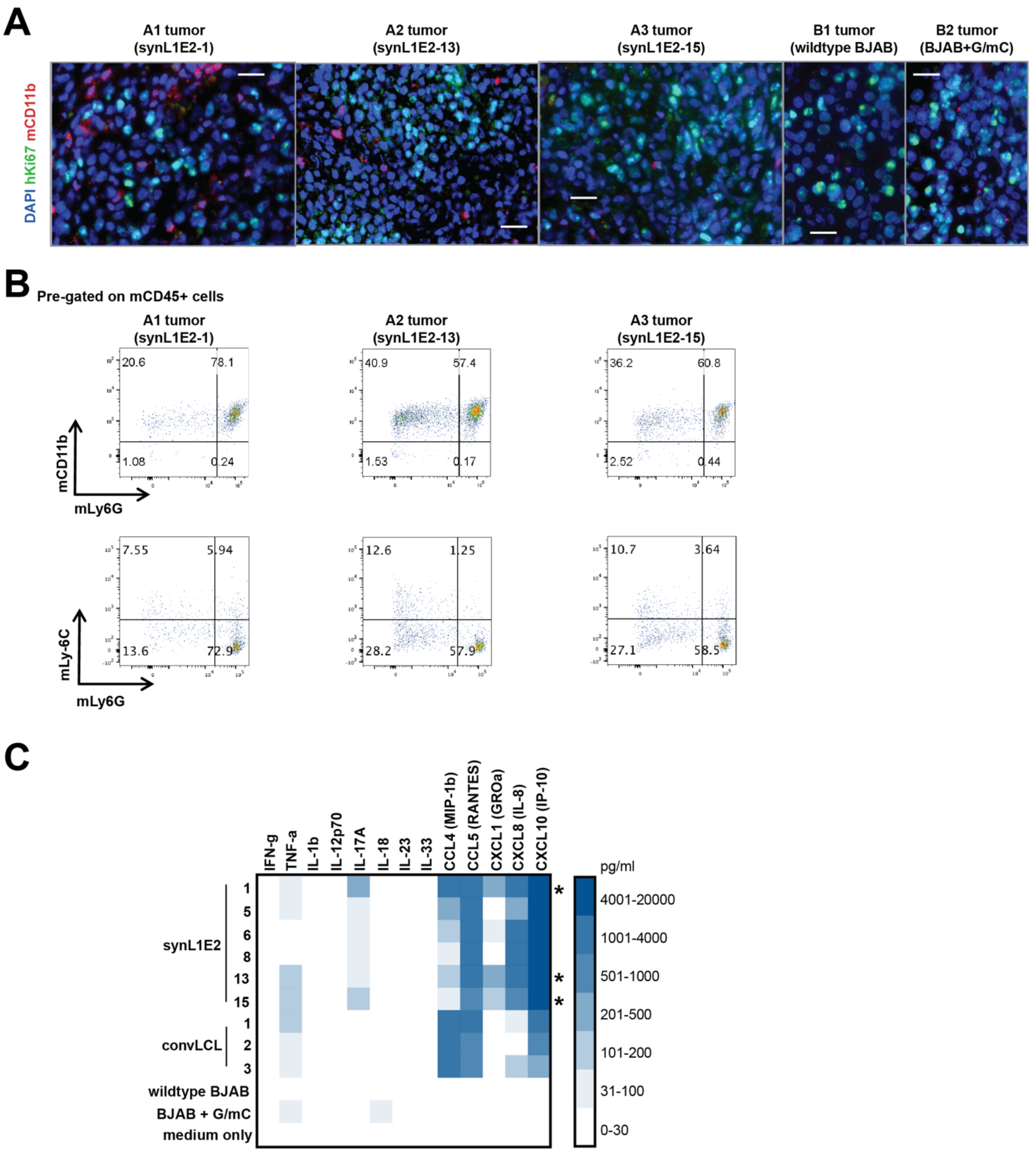
A
A1 tumor
(synL1E2-1)
A2 tumor
(synL1E2-13)
A3 tumor
(synL1E2-15)
B1 tumor
(wildtype BJAB)
B2 tumor
(BJAB+G/mC)
DAPI hKi67 mCD11b
B
Pre-gated on mCD45+ cells
A1 tumor
(synL1E2-1)
A2 tumor
(synL1E2-13)
A3 tumor
(synL1E2-15)
20.6
78.1
1.08
0.24
40.9
57.4
1.53
0.17
36.2
60.8
2.52
0.44
mCD11b
mLy6G
7.55
5.94
13.6
72.9
12.6
1.25
28.2
57.9
10.7
3.64
27.1
58.5
mLy-6C
mLy6G
C
IFN-g
TNF-a
IL-1b
IL-12p70
IL-17A
IL-18
IL-23
IL-33
CCL4 (MIP-1b)
CCL5 (RANTES)
CXCL1 (GROa)
CXCL8 (IL-8)
CXCL10 (IP-10)
synL1E2
1
5
6
8
13
15
convLCL
1
2
3
wildtype BJAB
BJAB + G/mC
medium only
*
*
*
pg/ml
4001-20000
1001-4000
501-1000
201-500
101-200
31-100
0-30

Supplementary Figure 7 (Supporting data for **Figure 4**). **(A)** Immunofluorescence of fixed and permeabilized cryosections from one synL1E2 tumor and two BJAB tumors (Experiment 2). Sections were stained with DAPI (blue; nuclei), anti-human Ki-67 (green; dividing cells) and anti-mouse CD11b (myeloid cells; red). Size bars, 20 µm. **(B)** Flow cytometry-based quantitation of CD45-positive mouse cells expressing Ly-6G in combination with CD11b (upper row; neutrophils) or Ly-6G in combination with Ly6C (lower row; neutrophils/monocytes) in three synL1E2 tumors from experiment 2. Cells were pre-gated on mCD45-positive cells. **(C)** Profiling of secreted pro-inflammatory cytokines and chemokines for six independent synL1E2 cell lines and three convLCLs derived from three EBV-negative donors. Wildtype BJAB cells, and BJAB cells expressing GFP and mCherry reporters ("BJAB + G/mC") were used for comparison. Asterisks mark the synL1E2 cell lines that were used in the *in vivo* experiments shown in **Figure 4**.

End of Supplementary Materials
